# Unsupervised clustering analysis of trauma/non-trauma centers using hospital features including surgical care

**DOI:** 10.1371/journal.pone.0306299

**Published:** 2024-08-22

**Authors:** Xiaonan Sun, Shan Liu, Charles Mock, Monica Vavilala, Eileen Bulger, Rebecca G. Maine

**Affiliations:** 1 Department of Industrial and Systems Engineering, University of Washington, Seattle, Washington, United States of America; 2 The University of Washington Department of Surgery, Harborview Medical Center, Seattle, Washington, United States of America; 3 Harborview Injury Prevention and Research Center, Seattle, Washington, United States of America; 4 The University of Washington Department of Anesthesia, Harborview Medical Center, Seattle, Washington, United States of America; North Memorial: North Memorial Health, UNITED STATES OF AMERICA

## Abstract

**Background:**

Injuries are a leading cause of death in the United States. Trauma systems aim to ensure all injured patients receive appropriate care. Hospitals that participate in a trauma system, trauma centers (TCs), are designated with different levels according to guidelines that dictate access to medical and research resources but not specific surgical care. This study aimed to identify patterns of injury care that distinguish different TCs and hospitals without trauma designation, non-trauma centers (non-TCs).

**Study design:**

We extracted hospital-level features from the state inpatient hospital discharge data in Washington state, including all TCs and non-TCs, in 2016. We provided summary statistics and tested the differences of each feature across the TC/non-TC levels. We then conducted 3 sets of unsupervised clustering analyses using the Partition Around Medoids method to determine which hospitals had similar features. Set 1 and 2 included hospital surgical care (volume or distribution) features and other features (e.g., the average age of patients, payer mix, etc.). Set 3 explored surgical care without additional features.

**Results:**

The clusters only partially aligned with the TC designations. Set 1 found the volume and variation of surgical care distinguished the hospitals, while in Set 2 orthopedic procedures and other features such as age, social vulnerability indices, and payer types drove the clusters. Set 3 results showed that procedure volume rather than the relative proportions of procedures aligned more, though not completely, with TC designation.

**Conclusion:**

Unsupervised machine learning identified surgical care delivery patterns that explained variation beyond level designation. This research provides insights into how systems leaders could optimize the level allocation for TCs/non-TCs in a mature trauma system by better understanding the distribution of care in the system.

## Introduction

Injuries are a major health concern, causing 16,000 deaths per day and over 5 million deaths per year worldwide [1,2]. In the United States, injury is the primary cause of death for individuals ≤ 44 years [1,2]. Trauma systems are an organized, multidisciplinary response to injuries of different severities across a geographic region, and have reduced mortality and improved injury outcomes [3,4]. Trauma systems aim to deliver timely appropriate care to all injured people within a geographic area. This is achieved by setting standards for the type of injury care different hospitals in the system should provide [5]. In trauma systems, hospitals are designated as trauma centers (TCs) and non-trauma centers (non-TCs). TCs within the United States are verified by the American College of Surgeons (ACS) and/or state departments of health as level I-V based on resources, trauma volume, and educational and research commitment [6]. Level I and level II TCs can provide trauma expertise, subspecialized care, and, frequently, more advanced technology than TCs with lower levels [7]. The key difference between level I and level II TCs is that level I TCs are high-volume teaching hospitals that engage in research and community outreach and serve as leaders within the trauma system [8], while Level II centers provide the same level of care for most injured patients, including the most severely injured, but may not have all highly specialized services and do not necessarily engage in the same research and community outreach [8]. Level Ⅲ, Ⅳ, and V TCs can provide definitive care for more minor injuries, and stabilize severely injured patients and transfer them to higher level TCs if needed [9]. Trauma system development has occurred mainly at the state level, and most states have legislation that designates TCs within the state hospital networks [9]. In Washington State, there is one level I TC, seven level II TCs, 73 lower-level TCs (III-V), and 19 non-TCs [8].

While the designation of TC levels sets rigorous requirements to which the TCs must adhere to ensure injured patients are treated at the most appropriate level of care, detailed trauma care delivery patterns are unexplored at different levels TCs in the practice [10]. For example, how the distribution of injuries and associated procedures should vary by TC levels is not specified in current guidelines. In an ideal system, patients would be treated at the lowest TC level that could care for their injury pattern and severity well, however, studies have shown that many patients are transferred for reasons other than medical necessity [11]. This can strain limited resources at higher level trauma centers, potentially decreasing their ability to care for the most severely injured patients [11,12]. Demographic characteristics of the patients, staffing issues, and economic factors are cited as reasons apart from medical needs associated with the transfer of injured patients to a higher-level TC [11]. Additional socioeconomic factors, such as payer type, social vulnerability, and area-level deprivation, may account for differences in trauma care within a trauma system [13]. Current trauma guidelines do not outline specific care that each level TC should provide or which injuries are best treated at a given level TC, and few studies have characterized the actual trauma care delivered among TC levels across a system [14,15]. Thus, gaining a better understanding the real-life variability in trauma care provided by different level TCs and non-TCs is vital to developing strategies to optimize trauma care in a state or region. Optimally aligning TCs functions with designation level and location, could decrease the risk of morbidity and mortality, under-triage, over-triage, and medical resource waste [8].

To explore the role that different level TCs play in a mature trauma system, we evaluated whether hospital features including surgical care delivered for injuries can distinguish hospitals by TC levels. We explored whether a cluster analysis of the TCs/non-TCs using these features would align with TC designation levels. Misalignment between clusters and TC levels would demonstrate variations in the type of care provided among TCs of the same designation level within a trauma system, implying that because of the multiple roles that hospitals play in the health care system, TC designation level may not be the only factor to consider when developing trauma system policies. Without a better understanding of this real-world variability in the delivery of trauma care by different centers, trauma system leaders cannot appropriately plan and allocate resources.

## Methods

### Data source

We assembled TC/non-TC features from the hospital discharge dataset in the Comprehensive Hospital Abstract Reporting System (CHARS). CHARS collects information for all inpatient admission for all WA state hospitals [16]. We used the dataset in the year 2016 for this study. We accessed the data on 21^st^ July, 2020 for research purposes, and the information we accessed could not identify individual participants. We matched hospital names and TC level designations according to the WA State Department of Health Trauma Services’ definition to all acute care hospitals [17]. We excluded all rehabilitation units, psychiatric units, and swing-bed units. All acute care hospitals that have not undergone state trauma level verification were considered non-TCs.

### Surgical care features

We classified the ICD-10-PCS procedure codes into four categories—minor diagnostic, minor therapeutic, major diagnostic, and major therapeutic, based on the Healthcare Cost and Utilization Product (HCUP)–US [18]. In this study, we only focused on the major therapeutic procedures (MPs), which are procedures typically performed in an operating room and performed for therapeutic reasons (e.g., open fractures fixation) [18] by the Diagnosis Related Group (DRG) [19].

### Procedure subgroup

Using the HCUP procedure categories [20] and expert review, we grouped the MPs into six subgroups: General Surgery, Orthopedics, Neurosurgery, Urology, Subspecialty (plastic surgery, obstetrics and gynecology, ophthalmology, etc.), and Other procedures. For similar procedures, we did not distinguish separate laterality (i.e., left or right side) and specific digit information. Because ICD-10 codes do not easily capture procedure complexity, we created procedure complexity groups (PCGs) based on injury and procedure.

### Procedure Complexity Group (PCG)

We assumed a positive correlation between injury severity and procedure complexity; therefore, we linked each procedure to injury severity in the related body region. To start, we generated a list of critical and frequent surgical procedures performed for injuries (see SA.1) and categorized ICD-10 procedure codes into each group. We used Abbreviated Injury Scale (AIS) to measure the injury severity of the body region. The AIS is an anatomical-based coding system created by the Association for the Advancement of Automotive Medicine to classify and describe the severity of injuries [21]. AIS scores were generated by the R package used to calculate Injury Severity Score (ISS) [22]. Unconverted diagnosis codes were matched using the American Automotive Association file, when possible [23]. Each procedure during a patient’s admission was assigned a PCG in the following format: common procedure category, related body region, and AIS score of the related body region (e.g., Open fixation, Extremities lower, 1).

### Other features

Other features include the proportion of patients who were male, median age, whether patients were admitted for trauma, transfer status, insurance payer type, ISS, injury mechanism, and social indices.

### Admission type

We classified each admission as trauma or non-trauma using the ICD-10-CM diagnosis codes [24]. Patients with at least one admission diagnosis classified as an injury in the National Trauma Data Standard (NTDS) were considered trauma patients [25]. Non-trauma admissions had no admission diagnoses included in the NTDS.

### Transfer type

Admissions were considered transfer-in if the patient was admitted from another acute care facility. Admissions were transfer-out if the patient was discharged to a different acute care facility. See SA-1 Table in S1 File for details of which facilities were included in acute care.

### Insurance payer type

We categorized the primary payer as private, low-income, or other payers. Private payers include health maintenance organization, commercial insurance, labor and industries, or health care service contractor. Low-income payers include Medicaid, self-pay, or charity care. The remaining types are other payers, which included Medicare.

### Injury Severity Score (ISS)

ISS is used to assess trauma severity and correlates with mortality, morbidity, and hospitalization time after trauma [26]. We first calculated ISS using the diagnosis codes for all trauma admissions and an ISS calculation R package [22]. Some diagnosis codes could not be converted to ISS components with the R package (SA-2 Table in S1 File), potentially underestimating the ISS, thus for some patients this value represents a minimum ISS.

### Injury mechanism

We classified the injury mechanism as blunt, penetrating, burn, or other for all trauma admissions by the principal E-Codes [27].

### Social indices

To include information about the socioeconomic status of both patients and the areas surrounding the hospital we calculated two separate indices. We used the Social Vulnerability Index (SVI) [28] for patients’ home residences, based on zip codes, and Social Deprivation Index (SDI) [29] for hospitals’ locations. SA.2 contains the calculation details and index mapping.

### Analysis

To explore whether care patterns aligned with TC level, we carried out statistical analyses on surgical care for injuries and other features for the hospitals by TC level. We tested whether there is a statistically significant variation for each feature across the TC levels using the chi-square and the Kruskal-Wallis as appropriate by distribution. A p-value ≤ 0.05 was considered significant. For features that included non-median counts, we performed an outlier detection test, and considered values >1.5 times the interquartile range (IQR) to be significant outliers.

We conducted 3 separate clustering analyses on TCs/non-TCs that performed MPs for trauma care. These three clustering analyses used different characteristics of the hospitals to provide complementary viewpoints on factors associated with different clustering. Hospital features not related to clinical care (e.g., percent of admissions that were for trauma) were consistent across the analyses. Table 1 describes the selected features in the three sets of clustering analyses. For each feature used, the percentage of missing values was at most 10% of admissions. All missing values were excluded from the analyses.

**Table 1:** Associated features in the three sets of clustering analyses.

	Set 1	Set 2	Set 3–1	Set 3–2
Name	Surgical specialty procedure subgroup labels and other features clustering	Surgical care PCG distribution and other features clustering	Surgical care volume clustering	Surgical care distribution clustering
**# TC/non-TC included**	69	53	53	53
**Threshold of # MP carried out for trauma admissions at each hospital for inclusion**	≥ 1	≥ 50	≥ 50	≥ 50
**Features**				
Surgical				
# MP	X	X		
% Subgroup MP	X	X		
PCG volume			X	
Clustering of # each PCG in Major General Surgery	X			
Clustering of # each PCG in Major Orthopedics	X			
Clustering of # each PCG in Major Neurosurgery	X			
Clustering of # each PCG in Major Urology	X			
Clustering of # each PCG in Major Subspecialty	X			
% PCG		X		X
Other (sex, age, admission type, transfer status, insurance payer type, ISS, injury mechanism, and social indices)	X	X		

Abbreviations: MP (major therapeutic procedures); PCG (Procedure Complexity Group), ISS (Injury Severity Score).

Given the number and breadth of procedures performed at each facility and across specialties, in the first analysis we sought to simplify how surgical care was included. In Set 1, we created a surgical care sub-cluster label for each surgical specialty using volumes of all unique MP performed within that specialty. The clustering by specialty identified broad patterns in specialty surgical care and reduced dimensionality. In Set 2 we considered the relative frequency of each PCG separately for TCs/non-TCs that had higher annual volumes for MPs performed for trauma care. We explored whether specific individual procedures contributed to the TCs/non-TCs separation when analyzed with the other hospital features. Detailed features for Set 1 and Set 2 are listed in SA-3 Table in S1 File. Finally, to determine whether only the differences in the type of surgical care performed for injuries can distinguish trauma center level, we conducted a third analysis using only the PCGs without other hospital features; resulting in two sub-analyses—surgical care volume clustering (Set 3–1) and surgical care distribution clustering based on MP frequency (Set 3–2). Originally, we had 438 PCGs, which we grouped by body region severity score (major injury: AIS ≥2.5; minor injury: AIS <2.5). We conducted the clustering analysis on these 130 modified PCGs. Instead of exploring how the overall volume of procedures within a specialty distinguishes the TCs/non-TCs as in Set 1, Set 3–1 examined how individual procedure volume impacts the TC/non-TC clusters. Set 3–2 explored whether procedure frequencies influence TC/non-TC separation without other features.

For each clustering analysis, we standardized the included features and conducted Principal Component Analysis (PCA). We selected the top components reaching 90% of the total variation from the PCA [30] and conducted an unsupervised clustering analysis using the Partition Around Medoids (PAM) [31] method. See details in SA.3. We summarized the contributing features for each analysis and determined if the clusters aligned with TC level designation.

## Results

In 2016, there were 34,645 trauma admissions and 601,328 non-trauma admissions across all hospitals in WA. SB-1 Table in S1 File shows a summary of surgical care and other hospital features/characteristics for all WA TCs/non-TCs by TC level.

### Set 1. Surgical care procedure subgroup labels and other features clustering

Of the 69 TC and non-TC that performed at least one MP for a trauma admission in 2016, there were 1 (100%) level I, 7 (100%) level II, 24 (100%) level Ⅲ, 26 (74%) level Ⅳ, 2 (14%) level V TCs and 9 (47%) non-TCs. For the procedure subgroup clustering, we chose the optimal cluster number to be between 3 and 10 clusters (SB-2 Table in S1 File). Subgroup clustering results are shown in SB-1 to SB-5 Figs in S1 File. In each specialty, the level I TC was in its own cluster, and higher-level TCs tend to have a higher volume of procedures. Procedure volume compared to other features primarily distinguished the TCs/non-TCs in each subgroup clustering.

**Fig 1:**
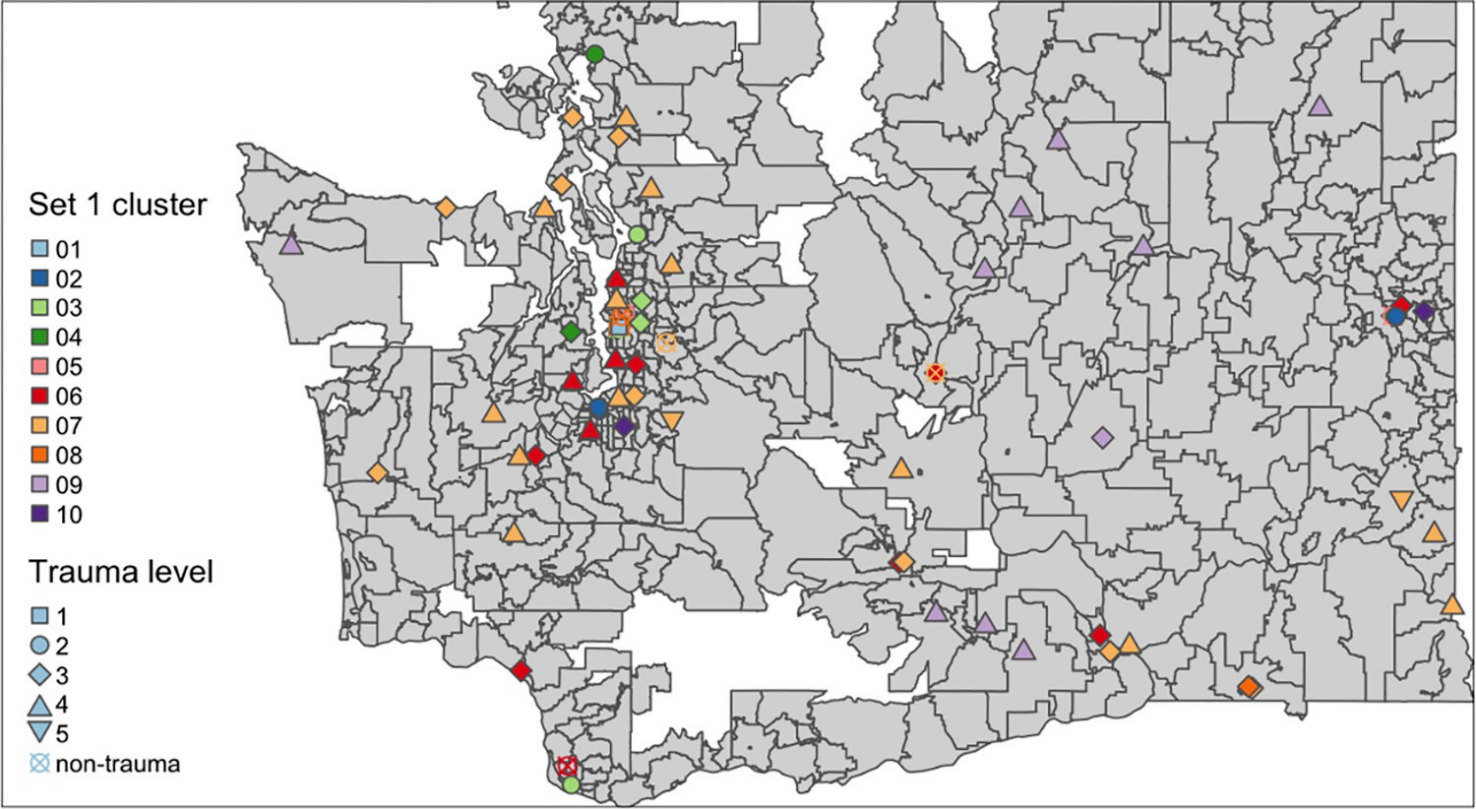
Clustering results displayed on a map of WA: Set 1 surgical care procedure subgroup labels and other features clustering. Note: The background of the map illustrates the division of zip codes (which was also used as the division of social indices calculation) within the state of Washington (WA). Each symbol on the map represents a hospital, where the geographic location is indicated by the symbol’s placement. The color of the symbol represents the cluster to which the hospital belongs, and the shape denotes the designated trauma level.

**Fig 2:**
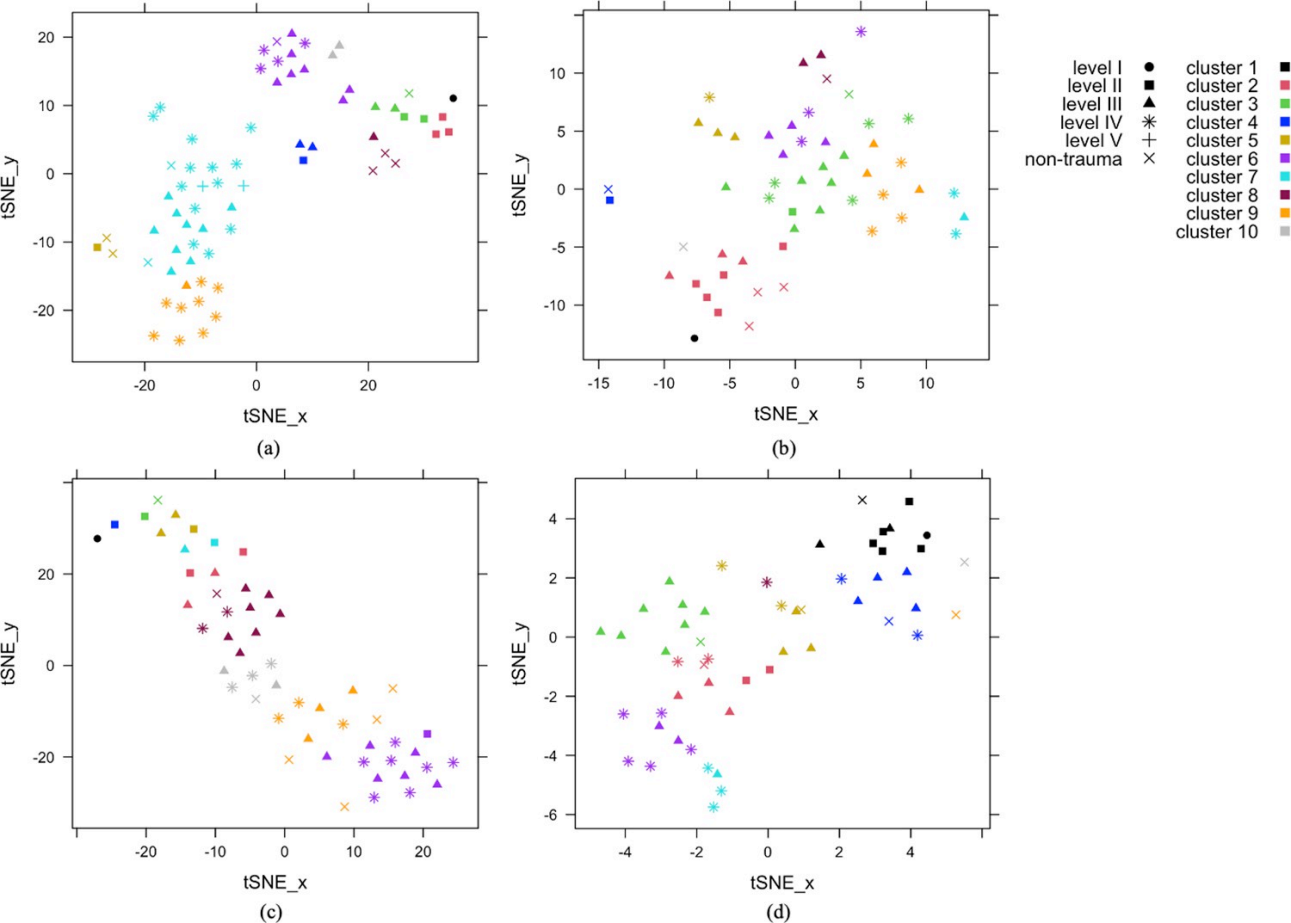
Clustering results displayed on a 2-dimensional space: (a) Set 1 surgical specialty procedure subgroup labels and other features clustering, (b) Set 2 surgical care PCG distribution and other features clustering, (c) Set 3–1 surgical care volume clustering, (d) Set 3–2 surgical care distribution clustering. Note: Each symbol represents a hospital, with the distance indicating the relative distances based on all the clustering features. Hospitals with high-dimensional data for all features are visualized using the t-SNE method, which assigns each data point a location in a two-dimensional space, mapping similar data points closely together. Other features include sex, age, admission type, transfer status, insurance payer type, ISS, injury mechanism, and social indices.

In the combined Set 1 analysis, we kept features that accounted for 90% of the total variance captured by the top 10 principal components (SB-6 Fig in S1 File). Comparing the PAM clustering results with the number of clusters ranging from 1 to 10, we chose 10 clusters to obtain a near-optimal clustering performance with the most separation (SB-7 Fig in S1 File). Figs 1 and 2(A) show the visualized clustering results. SB-3 Table in S1 File gives the summary of all the original features that contributed to the clusters. It also highlights the key features that contributed at least 10% of the variation in the top 3 principal components, which themselves explained 60% [32] of the total variance. Table 2 presents the summary of these key features. The level I TC was alone in cluster 1, cluster 2 contained only level II TCs, cluster 10 contained only level Ⅲ TCs, and the other clusters contained a mix of different TC levels. The three pediatric hospitals were in cluster 5. PCG cluster labels from procedure subgroups are the major contributors compared to other features. This implies that beyond the total volume of surgical care, the volume for each specialty also varies and distinguishes TCs/non-TCs even among hospitals of the same level. For example, level Ⅲ TCs in cluster 10 and cluster 8 provided a similar amount of trauma MP of all specialties, while the ones in cluster 10 provided more orthopedic care and less neurosurgery care (see SB-3 Table in S1 File).

**Table 2:** Key features contributed to the TCs/non-TCs clusters from Set 1 surgical care procedure subgroup labels and other features clustering.

**Cluster**	1	2	3	4	5	6	7	8	9	10
**TC/non-TC levels in the cluster**	Ⅰ	Ⅱ	Ⅱ, Ⅲ, Non	Ⅱ, Ⅲ	Ⅱ, Non	Ⅲ, Ⅳ, Non	Ⅲ, Ⅳ, Ⅴ, Non	Ⅲ, Non	Ⅲ, Ⅳ	Ⅲ
**# TC/non-TC in the cluster**	1	3	5	3	3	12	26	4	10	2
**Cluster mean**										
% TC/non-TC in General Surgery label 2^a^	0	100%*	80%	100%	0	0	0	75%	0	0
% TC/non-TC in General Surgery label 3^a^	0	0	20%	0	100%	100%	100%	25%	100%	100%
% TC/non-TC in Orthopedics label 2^a, b^	0	100%	100%	100%	0	100%	0	0	0	100%
% TC/non-TC in Orthopedics label 3^a, b^	0	0	0	0	100%	0	100%	100%	100%	0
% TC/non-TC in Neurosurgery label 2^a^	0	100%	100%	0	0	17%	0	75%	0	0
% TC/non-TC in Neurosurgery label 3^a^	0	0	0	100%	100%	83%	100%	25%	100%	100%
% TC/non-TC in Urology label 3^c^	0	0	80%	0	0	0	0	75%	0	100%
% TC/non-TC in Urology label 4^a^	0	0	0	100%	100%	100%	92%	25%	100%	0

a: Features that contribute no less than 10% of the variation within the 1^st^ principal component.

b: Features that contribute no less than 10% of the variation within the 2^nd^ principal component.

c: Features that contribute no less than 10% of the variation within the 3^rd^ principal component.

*: 100% percent of the TCs/non-TCs in cluster 1 are with general surgery label 1.

Abbreviations: TC (Trauma Center); MP (major therapeutic procedures).

### Set 2. Surgical care PCG distribution and other features clustering

Of the 53 TC and non-TC that performed ≥ 50 MPs for trauma in 2016, there were 1 (100%) level I, 7 (100%) level II, 23 (96%) level Ⅲ, 15 (43%) level Ⅳ, 0 level Ⅴ, and 7 (37%) non-TCs. The top 9 principal components, which explained 90% of the variance in the PCA, were included (SB-9 Fig in S1 File). Comparing the PAM clustering results with the number of clusters ranging from 1 to 10, we also chose 10 clusters (SB-10 Fig in S1 File). Figs 2(B) and SB-8(a) in S1 File show the visualized clustering results. SB-4 Table in S1 File gives the summary of all the original features that contributed to the clusters, again highlighting the features that contributed at least 10% of the variation within the top 4 principal components, which was the number of principal components that explained 60% [32] of the total variance that drove TC/non-TC separation. Table 3 presents the summary focusing only on these key features.

**Table 3:** Key features contributed to the TCs/non-TCs clusters from Set 2 surgical care PCG distribution and other features clustering.

**Cluster**	1	2	3	4	5	6	7	8	9	10
**TC/non-TC levels in the cluster**	Ⅰ	Ⅱ, Ⅲ, Non	Ⅱ, Ⅲ, Ⅳ, Non	Ⅱ, Non	Ⅲ, Ⅳ	Ⅲ, Ⅳ	Ⅲ, Ⅳ	Ⅲ, Non	Ⅲ, Ⅳ	Non
**# TC/non-TC in the cluster**	1	11	14	2	4	7	3	3	7	1
**Cluster mean**										
Median age of trauma patients (year)^b^	47	65	72	9	70	71	69	75	74	72
Median age of non-trauma patients (year)^b, c^	55	54	56	6	38	52	60	43	62	66
# Trauma admissions^d^	5605	1042	547	270	434	225	188	784	235	495
SDI in TC/non-TC area^c, d^	74	82	46	62	63	66	56	24	41	61
Mean SVI in trauma patient residence^c^	0.51	0.53	0.50	0.53	0.59	0.66	0.63	0.30	0.43	0.40
Mean SVI in non-trauma patient residence^c^	0.54	0.55	0.51	0.52	0.60	0.66	0.62	0.30	0.45	0.44
% Non-trauma patients with private payer^c^	22%	34%	35%	42%	61%	20%	40%	56%	28%	31%
# Non-trauma MP^d^	8730	11912	4401	4438	3997	1279	3130	8309	1310	11040
% Trauma major Orthopedics in all MP^a^	53%	62%	81%	62%	84%	92%	65%	77%	90%	2%

a: Features that contribute no less than 10% of the variation within the 1^st^ principal component.

b: Features that contribute no less than 10% of the variation within the 2^nd^ principal component.

c: Features that contribute no less than 10% of the variation within the 3^rd^ principal component.

d: Features that contribute no less than 10% of the variation within the 4^th^ principal component.

Abbreviations: TC (Trauma Center); SDI (Social Deprivation Index); SVI (Social Vulnerability Index); MP (major therapeutic procedures); PCG (Procedure Complexity Group).

The result showed that the level I TC was alone in cluster 1, cluster 10 contained only one non-TC, and the other clusters contained a mix of different TC levels. Two pediatric hospitals were in cluster 4. The features that distinguished the clusters were primarily at the hospital level, rather than the specific type of trauma surgeries were performed. Out of all PCGs, only the orthopedic surgical care contributed to the resulting clusters. The nine features that contributed the most to clustering were mean patient age for trauma and non-trauma patients, number of trauma admissions, SDI of the hospital neighborhood, mean patient SVI for trauma and non-trauma patients, payer mix, number of non-trauma operations annually, and percent of trauma operations done for orthopedic injuries. The proportion of all MPs on trauma patients that were for orthopedic injuries contributed the most to the 1^st^ principal component. Age contributed greatly to the 2^nd^ principal component. Financial and social factors such as the proportion of non-trauma patients with a private payer, age of non-trauma patients, SDI in hospital areas, and SVI in the patient residences all contributed at least 10% to the 3^rd^ principal component. The volume of MPs for non-trauma patients and total trauma admissions contributed the most to the 4^th^ principal component. Several clusters with level II TCs and non-TCs had a higher proportion of younger patients and more private payers. Differences in SDI and SVI separated some of the TCs at the same levels into different clusters; clusters with high SDI did not necessarily have high SVI.

### Set 3. Surgical care clustering

The same 53 TCs and non-TCs from Set 2 were used in the Set 3 analysis. For Set 3–1 which only included surgical care volume clustering using PCGs, over 90% of the total variance came from the 1^st^ principal component (SB-11 Fig in S1 File). We chose 10 clusters to be directly comparable to the results from Set 2 (Figs SB-12, 2(c) and SB-8(b) in S1 File). The level I TC was, again, alone in cluster 1. Cluster 2 contained only one level II TC, and all other clusters contained a mix of different TC levels (SB-5 Table in S1 File). The TCs/non-TCs are generally separated by the total volume of major procedures conducted, and the clusters with greater total volume MPs also tend to perform a greater volume of each individual PCG. Among all the PCGs, the number of open fixations on the lower extremities contributed the most to the clusters, and the top contributing features were mostly orthopedic.

Rather than absolute volume, Set 3–2 focused on the case mix using the relative frequencies of each PCG for each hospital. In Set 3–2, 90% of the total variance was explained by 10 principal components (SB-13 Fig in S1 File). We clustered the 53 TCs and non-TCs into 10 clusters (Figs 2(D) and SB-8 (c) in S1 File). Four procedure groups accounted for at least 10% of the variation within the top 2 principal components, which captured over 60% of the total variance [32] (SB-6 Table in S1 File). This was the only analysis in which the level I TC was not in a unique cluster. The level I TC was clustered with eight other TCs and non-TCs including two pediatric hospitals. The major contributing features all belonged to orthopedic care. Level Ⅲ and level Ⅳ TCs varied in terms of the proportion of open fixations on lower extremities with a minor injury, and the cluster containing level I TC had a relatively small proportion of procedures on joints on lower extremities with a minor injury. Surgical procedures on the lower extremities had the greatest influence on the clusters, with the proportion of open fixation on lower extremities with a minor injury being the primary variable in the 1^st^ principal component. In the 2^nd^ principal component, the four procedures whose relative frequency contributed the most were all on the lower extremities: procedures on the joint in lower extremities with a minor injury, open fixation of a minor injury on lower extremities, and percutaneous fixation of both major and minor injuries in the lower extremities.

## Discussion

To the best of our knowledge, this is the first study attempting to understand the real-life variability of the surgical trauma care provided by different level TCs/non-TCs in a mature trauma system using machine learning applied to both surgical care and hospital-level features derived from patient-level admission and injury data. It highlights the promise of unsupervised machine learning to help trauma system leaders identify the needs and optimize the efficiency of trauma care delivery. In this study, our three cluster analyses of surgical care features and other hospital features found that the clusters only partially aligned with TC designation levels. This demonstrates that though hospitals may have equivalent trauma level designations, their care of injured patients may vary greatly. Given the multiple roles that TC play in providing health care to their communities, this finding is not surprising, however, it has implications for policymakers when considering how to improve trauma systems. The novelty of this work lies in four aspects. First, we classified surgical procedures for injury into novel procedure complexity groups that combined procedures with the injury severity of the body region to approximate the complexity of the procedures, which cannot be determined from ICD-10 codes alone. Second, we explored various ways to combine the surgical care features and other features in different analyses to better understand the relative contribution of each feature type to the clustering result. Third, while most studies investigate the impact of trauma or non-trauma care separately, our study included both trauma and non-trauma care features to evaluate how the mature trauma system functions as a whole. Fourth, we used unsupervised machine learning methods to demonstrate that TCs/non-TCs within the same designation level frequently do not share the same patient characteristics nor do they provide the same mix of surgical care.

Together, these three analyses provide a novel approach to gain insights into the distribution of trauma care in a mature trauma system. Most trauma systems, including in Washington State, are not developed de novo, but rather are built using existing hospitals whose purpose is to provide care for a multitude of problems, not just injuries. In addition, given the location of different centers, the population in the surrounding areas may have different characteristics that also impacted this analysis. Given the TC and non-TC frequently have multiple priorities in providing care for their communities and often have populations with similar characteristics (e.g., lower income, higher rates of uninsurance), it is not surprising that the cluster analysis did not find perfect alignment between hospitals of the same TC level. However, this analysis can provide insights that could help trauma system leaders identify which hospitals may be most easily changed within the trauma system to serve a different role and which hospital and surgical care features may be most important to consider when developing trauma system policies and addressing issues of equity in access to trauma care. First, for hospitals that performed at least one surgery for trauma in a year, the volume and types of surgical care are the primary drivers of the hospital clusters. Second, in the hospitals with higher volumes of operative trauma, the characteristics of the patient population (e.g., age of trauma and non-trauma admissions, payer mix) played a larger role in cluster formation than the specific type of trauma surgery they were performing. Finally, when only operative care was considered at hospitals with higher trauma surgical volumes (Set 3), the two analyses demonstrate that the volume of the procedure rather than the relative proportions of each type of procedure aligns more, though not completely, with TC designation. Set 3–2 used surgical care case mix, and it was the only analysis that did not separate the level I TC into a unique cluster, implying although level I TC conducts a greater volume of surgical procedures, the relative frequency of each PCG does not vary greatly from the other level TCs. Interestingly, the clustering of hospitals varied greatly with the inclusion of different factors, and surgical care was not always the largest contributor to the group differences.

This study included surgical care for both trauma and non-trauma patients as potential factors that would contribute to hospital clusters. We included this because while logically there is a potential relationship between capacity for trauma care and non-trauma conditions at a hospital, it has not been well evaluated in the literature. By including both groups, this analysis provides additional insight into how delivery of other types of surgical care relates to the surgical care for trauma care around the state’s trauma system as well as how trauma and non-trauma populations vary across hospitals. The age differences among the trauma patients aligned more with the TC designation compared to non-trauma patients treated at those hospitals. The level I TC served the youngest population, which aligns with previous research that younger people are more prone to have more severe injuries and thus be treated at a higher level of care [33], and that elderly patients are less likely to be transferred to a higher level of care for their injuries [34,35]. Note that little difference is shown in residence SVI for trauma and non-trauma admissions, which was surprising because we anticipated that trauma patients would differ from non-trauma patients since trauma patients frequently come from a wider referral area than other patients treated at the hospital. Some of the trauma centers are also centers of excellence for non-trauma conditions like strokes and myocardial infarctions [36], and the referral of patients from a larger catchment area for these conditions as well may have diminished any expected difference in the groups. In terms of the payer mix among the level Ⅲ TCs, some have a higher proportion of private or low-income payers for non-trauma patients only, and some have a lower proportion of private and low-income payers for trauma patients only, implying a relatively large proportion of trauma patients are with “other payers” which include Medicare. This analysis suggests that the inclusion of both trauma and non-trauma care are important when analyzing how to improve a trauma system, as non-trauma care also contributes to natural hospital clusters.

An underlying motivation of this work was to explore a means of analyzing a large data set to provide insight into the factors associated with TC level designation across a mature system that can then be used to inform policies to address equity and efficiency in trauma care delivery. We identified that the real-life variabilities in trauma care only partially aligned with the current trauma level designation. The volume of surgical procedures performed, especially orthopedic procedures, contributed to the differences among level Ⅲ TCs. The payer mix and social index also had an impact, which distinguished the TCs/non-TCs more by the location they serve rather than the designation level. Not surprisingly, SDI for hospitals and the SVI of the patients they treated were correlated. The level II TCs in the area with higher social deprivation tend to have patients living in more vulnerable residences and with a higher proportion of low-income payers compared to the level II TCs in the area with lower social deprivation, which implies patients tend to go to the closest hospital. Previous work has shown that TCs in the area with higher deprivation and vulnerability scores may receive fewer funds due to lower taxes and a less favorable payor mix and therefore have fewer resources, potentially leading to differences in care, outcomes, and efficiency for these centers [37]. This highlights potential for inequity since patients nationwide may not have equal access to high-quality trauma care services [38]. We also found that while in the majority of the TCs/non-TCs orthopedic surgical care was the largest proportion of overall surgical care, there were some TCs/non-TCs with a relatively small proportion of orthopedic care and a relatively large proportion of neurosurgery care. Trauma systems were developed with existing hospital infrastructures in most states and improving them is challenging because of resource limitations and few tools to guide improvement. To date, the existing tools do not incorporate the complexity of the interactions between multiple parts of the system [39]. This study shows potential directions to optimize trauma system functioning apart from adding new TCs or transport hubs, as machine learning provides insight into which TCs/non-TCs are the best candidates to be designated at a particular level based on the current operative trauma care and hospital features. However, TC designation does not encourage uniformity and homogeneity; its designation standards are meant to ensure a minimum standard. Beyond this standard, healthcare centers can innovate and adjust the care they provide using their available resources. This flexibility could be essential for hospitals to manage in the current economic situation. In this context, our study can be a valuable tool for understanding these variations, going beyond just focusing on allocation strategies.

This study has several limitations. Access to more recent state data was limited during COVID because of pressing public health needs, thus we limited our analysis to the available 2016 data. It is possible that this year of data is not representative of patterns of care in later years, and it limits our ability to assess changes over time or distinguish whether the observed cluster variations reflect a static phenomenon or an evolving trend influenced by selection pressures. However, even this one year of data demonstrates the potential of machine learning methods to evaluate the role of surgical care in distinguishing hospitals in a trauma system. This in turn offers promising insights into optimizing trauma systems and sets a foundation for future research to investigate trends over multiple years of data. CHARS uses discharge data, and there is a potential for coding errors in the data. In addition, not all the recorded diagnosis codes were successfully converted to ISS, which may introduce bias to our ISS calculation. Nevertheless, this bias happened across all TC levels and is less likely to impact the clustering patterns. While conducting this study in different state(s) could provide deeper insights into regional differences and identify overarching latent forces and behaviors, our research is solely focused on Washington State. Nonetheless, our study can serve as a reference for similar analyses in other states, promoting comparative research and broadening our understanding of trauma care dynamics on a larger scale. In terms of the procedures, the HCUP classification tools are not perfect and required manual reclassification. In the absence of standard recoding schemes, however, this improved the accuracy of our analysis. Furthermore, ICD procedure codes do not contain information about the injury’s complexity; again, a standard method of determining this has not been developed. As such, we internally developed PCGs to approximate procedure complexity. In addition, we elected to only include major procedures, to limit the number of variables in the analysis to those that likely require a higher level of expertise. Including the majority of procedures that might reasonably be expected to be available at most hospitals of any TC designation level would not distinguish well between the clusters. Looking at minor procedures, especially with a population that includes patients treated and discharged from the emergency room is a future direction of this work. Finally, this data set only included inpatient information. However, as we were focused on surgical care, this data set captured the relevant patient population. Integrating data from multiple sources, including the emergency departments, prehospital information, and inter-facility transfer information, is now needed to see if this can identify additional distinguishing features. Finally, these results reflect a single trauma system, and evaluation of other trauma systems using similar methods is also needed to validate for generalizability.

## Conclusions

This study analyzed the relationship between the real-life variability in trauma surgical care and state TC designation levels for hospitals in WA using three sets of clustering analyses. By demonstrating that operative trauma and non-trauma care are only partially aligned with the current TC designation level, this study shows that when considering trauma system improvements, not all hospitals with the same TC designation are equivalent in the actual care they provide. This study suggests unsupervised machine learning could be a promising method to offer insight into the contribution of different hospitals in a specific trauma system, helping to identify which hospitals are the optimal candidates for designation level change or the incorporation of additional services. This is crucial if tools to improve trauma systems are going to be developed that can consider a broader and more nuanced range of interventions that goes beyond simply changing the number and/or location of TCs.

## Supporting information

S1 File Supplemental methods and results. (DOCX)

## Abbreviations

TC: trauma-center
HCUP: Healthcare Cost and Utilization Product
MP: major therapeutic procedure
PCG: procedure complexity group
AIS: Abbreviated Injury Scale
ISS: Injury Severity Score
SVI: Social Vulnerability Index
SDI: Social Deprivation Index
PCA: Principal Component Analysis
PAM: Partition Around Medoids
