# Supplementary material for: Unsupervised clustering analysis of trauma/non-trauma centers using hospital features including surgical care
Source: PLoS One. 2024 Aug 22;19(8):e0306299. doi: 10.1371/journal.pone.0306299 (PMC11340941; doi:10.1371/journal.pone.0306299)
Supplement: S1 File — (DOCX) [file pone.0306299.s001.docx]

**Unsupervised Clustering Analysis of Trauma/Non-Trauma Centers Using Hospital Feature Including Surgical Care**

**Supporting information**

**S. Supplemental methods and results**

**SA. Supplemental methods**

**SA.1. PCG supplemental information**

Using our team’s trauma expertise, we grouped specific MPs into larger groups of common procedures performed for injuries (e.g., craniotomy, repair of open fracture of the upper extremity, etc.) (SA.1-1 Table), then linked these categories to the related body region and the AIS score for that body region. The body regions include the head, neck, face, upper extremities, lower extremities, chest, and abdomen. We obtained the maximum AIS score from the ISS calculation R package [1] for each of the six ISS body regions: head and neck, face, extremities, chest, abdomen, and general. For the diagnosis codes that are not successfully converted, we matched them with AIS codes provided by the coder in Harborview Medical Center, then identified the injury body regions and injury scores. The 1^st^ digit of the AIS code represents body region, which includes head, face, neck, thorax, abdomen, spine, upper extremity, lower extremity, and unspecified; the 7^th^ digit of the AIS code represents severity score, which includes 1,2,3,4,5,6, and 9 [2]. Since the AIS code is in more detail than the ICD-10-CM code, each diagnosis code may be related to more than one AIS code. To match what we obtained from the ISS calculation R package [1], we converted the AIS code body region as shown in SA.1-2 Table. If one diagnosis code is related to more than one severity score, we calculated the average to be the injury score of that code; If the score is 9 which is not further specified, we treated it as 0. Then we calculated the maximum AIS score for each of the six ISS body regions for admissions with unconverted diagnosis code(s). Since we do not know which diagnosis related to which procedure, we assigned the augmented body region maximum AIS scores accordingly to the PCGs as shown in SA.1-3 Table within the same admission to approximate the injury severity for each procedure.

SA.1-1 Table: Common procedure categories for PCGs

| **Category No.** | **Common procedure categories** |
| --- | --- |
| 1 | Amputation |
| 2 | Cardiac |
| 3 | Control of hemorrhage |
| 4 | Craniectomy |
| 5 | Craniotomy |
| 6 | Ex fix |
| 7 | Exploratory laparotomy/other abdominal surgery |
| 8 | Facial fractures |
| 9 | Gynecology |
| 10 | Joint |
| 11 | Neck exploration |
| 12 | Open fixation |
| 13 | Open pelvis fixation |
| 14 | Ophthalmology |
| 15 | Other ent |
| 16 | Other general surgery |
| 17 | Other neurosurgery |
| 18 | Other orthopedics |
| 19 | Other subspecialty |
| 20 | Other thoracic |
| 21 | Other urology |
| 22 | Other vascular procedures |
| 23 | Pelvis external fixation |
| 24 | Percutaneous fixation |
| 25 | Percutaneous pelvic fixation |
| 26 | Peripheral nerve |
| 27 | Reconstruction |
| 28 | Rib fixation |
| 29 | Spine procedures |

SA.1-2 Table: AIS conversion

| **1^st^ digit of the AIS code** | **AIS code body region** | **Conversion method** |
| --- | --- | --- |
| 1 | Head | Belongs to “head and neck” |
| 2 | Face | Belongs to “face” |
| 3 | Neck | Belongs to “head and neck” |
| 4 | Thorax | Belongs to “chest” |
| 5 | Abdomen | Belongs to “abdomen” |
| 6 | Spine | Manually checked each related diagnosis code and grouped |
| 7 | Upper extremity | Belongs to “extremities” |
| 8 | Lower extremity | Belongs to “extremities” |
| 9 | Unspecified | Belongs to “general” |

SA.1-3 Table: PCG body region AIS severity score assignment

| **PCG body region** | **Assigned AIS score** |
| --- | --- |
| Head | Maximum “head and neck” AIS score |
| Neck | Maximum “head and neck” AIS score |
| Face | Maximum “face” AIS score |
| Upper extremities | Maximum “extremities” AIS score |
| Lower extremities | Maximum “extremities” AIS score |
| Chest | Maximum “chest” AIS score |
| Abdomen | Maximum “abdomen” AIS score |

SA-1 Table: Transfer-out status list

| **Code** | **Code value** |
| --- | --- |
| 1 | Discharged to home/self care (routine charge). |
| 2 | Discharged/transferred to other short term general hospital for inpatient care. |
| 3 | Discharged/transferred to skilled nursing facility (SNF) with Medicare certification in anticipation of covered skilled care -- (For hospitals with an approved swing bed arrangement, use Code 61 - swing bed. For reporting discharges/transfers to a non-certified SNF, the hospital must use Code 04 - ICF. |
| 4 | Discharged/transferred to intermediate care facility (ICF). |
| 5 | Discharged/transferred to another type of institution for inpatient care (including distinct parts). NOTE: Effective 1/2005, psychiatric hospital or psychiatric distinct part unit of a hospital will no longer be identified by this code. New code is '65' |
| 6 | Discharged/transferred to home care of organized home health service organization. |
| 7 | Left against medical advice or discontinued care. |
| 8 | Discharged/transferred to home under care of a home IV drug therapy provider. (discontinued effective 10/1/05) |
| 9 | Admitted as an inpatient to this hospital (effective 3/1/91). In situations where a patient is admitted before midnight of the third day following the day of an outpatient service, the outpatient services are considered inpatient. |
| 20 | Expired (did not recover - Christian Science patient). |
| 21 | Discharged/transferred to Court/Law Enforcement (eff. 10/2009) |
| 30 | Still patient or expected to return for outpatient services |
| 40 | Expired at home (hospice claims only) |
| 41 | Expired in a medical facility such as hospital, SNF, ICF, or freestanding hospice. (Hospice claims only) |
| 42 | Expired - place unknown (Hospice claims only) |
| 43 | Discharged/transferred to a federal hospital (eff. 10/1/03) |
| 50 | Hospice - home (eff. 10/96) |
| 51 | Hospice - medical facility (eff. 10/96) |
| 61 | Discharged/transferred within this institution to a hospital-based Medicare approved swing bed (eff. 9/01) |
| 62 | Discharged/transferred to an inpatient rehabilitation facility including distinct parts units of a hospital. (eff. 1/2002) |
| 63 | Discharged/transferred to a long term care hospitals. (eff. 1/2002) |
| 65 | Discharged/Transferred to a psychiatric hospital or psychiatric distinct unit of a hospital (these types of hospitals were pulled from patient/discharge status code '05' and given their own code). (eff. 1/2005). |
| 66 | Discharged/transferred to a Critical Access Hospital (CAH) (eff. 1/1/06) |
| 69 | Discharged/transferred to a designated disaster alternative care site (eff. 10/2013) |
| 70 | Discharged/transferred to another type of health care institution not defined elsewhere in code list. |
| 71 | Discharged/transferred/referred to another institution for outpatient services as specified by the discharge plan of care (eff. 9/01) (discontinued effective 10/1/05) |
| 81 | Discharged to home or self-care with a planned acute care hospital readmission (eff. 10/2013) |
| 82 | Discharged/transferred to a short term general hospital for inpatient care with a planned acute care hospital inpatient readmission (eff. 10/2013) |
| 83 | Discharged/transferred to a skilled nursing facility (SNF) with Medicare certification with a planned acute care hospital inpatient readmission (eff. 10/2013) |
| 84 | Discharged/transferred to a facility that provides custodial or supportive care with a planned acute care hospital inpatient readmission (eff. 10/2013) |
| 85 | Discharged/transferred to a designated cancer center or children’s hospital with a planned acute care hospital inpatient readmission (eff. 10/2013) |
| 86 | Discharged/transferred to home under care of organized home health service organization with a planned acute care hospital inpatient readmission (eff. 10/2013) |
| 87 | Discharged/transferred to court/law enforcement with a planned acute care hospital inpatient readmission (eff. 10/2013) |
| 88 | Discharged/transferred to a federal health care facility with a planned acute care hospital inpatient readmission (eff. 10/2013) |
| 89 | Discharged/transferred to a hospital-based Medicare approved swing bed with a planned acute care hospital inpatient readmission (eff. 10/2013) |
| 90 | Discharged/transferred to an inpatient rehabilitation facility (IRF) including rehabilitation distinct part units of a hospital with a planned acute care hospital inpatient readmission (eff. 10/2013) |
| 91 | Discharged/transferred to a Medicare certified long term care hospital (LTCH) with a planned acute care hospital inpatient readmission (eff. 10/2103) |
| 92 | Discharged/transferred to nursing facility certified under Medicaid but not certified under Medicare with a planned acute care hospital inpatient readmission (eff. 10/2013) |
| 93 | Discharged/transferred to a psychiatric hospital/distinct part unit of a hospital with a planned acute care hospital inpatient readmission (eff. 10/2013) |
| 94 | Discharged/transferred to a critical access hospital (CAH) with a planned acute care hospital inpatient readmission (eff. 10/2013) |
| 95 | Discharged/transferred to another type of health care institution not defined elsewhere in this code list with a planned acute care hospital inpatient readmission (eff. 10/2013) |

SA-2 Table: Trauma diagnosis codes that cannot be converted

| **No.** | **ICD10CM Code** |
| --- | --- |
| 1 | S62024B |
| 2 | S63435A |
| 3 | S82245C |
| 4 | S90529A |
| 5 | S95292A |
| 6 | T22641A |
| 7 | T22649A |

**SA.2. Social indices supplemental information**

We used the Social Vulnerability Index (SVI) [3] for patients’ home residences and Social Deprivation Index (SDI) [4] for hospitals’ locations. Social vulnerability is derived using several factors including poverty, lack of access to transportation, and crowded housing that may weaken a community’s ability to prevent human suffering and financial loss in a disaster [3]. The SVI ranks each census tract on these social factors based on percentiles with values ranging from 0 to 1, with higher values indicating greater vulnerability [3]. The SDI was developed to quantify levels of disadvantage in income, education, housing, transportation, and employment across small areas, evaluate their associations with health outcomes, and address health inequities [4]. The SDI ranges from 0 to 100 for each census tract with higher scores indicating higher deprivation [4]. We linked the zip codes of the patient residences and hospital addresses to census tracts [5], then averaged the SVI and SDI across the census tracts in each zip code. We included social indices to explore the relationship between the SDI of TCs/non-TCs location and SVI of the patient residence and we expect that patients living in areas with higher SVI tend to go to TCs/non-TCs located in the area with higher SDI. We calculated the patient SVI for each hospital as the average SVI among all the patient residence zip codes. SA.2-1 Fig maps the SDI of hospital zip codes with average patient SVI in TCs/non-TCs in WA state and zooms in on King County, Snohomish County, and Pierce County. SA.2-2 Fig includes only level I, II, and III TCs in WA state.


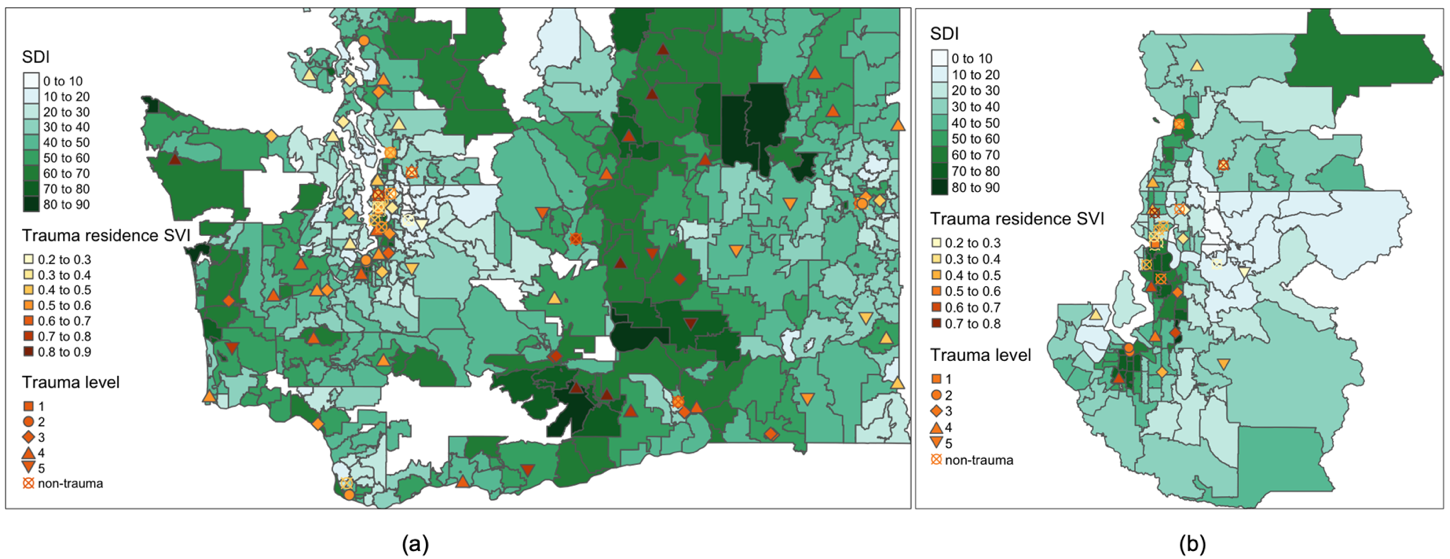


Each symbol represents a TC/non-TC.

Average patient SVI: calculated as the average SVI among all the patient residence zip codes for each TC/non-TC.

SA.2-1 Fig: (a) SDI of hospital zip code with average patient SVI in TCs/non-TCs in WA state

(b) SDI of hospital zip code with average patient SVI in TCs/non-TCs in King County, Snohomish County, and Pierce County


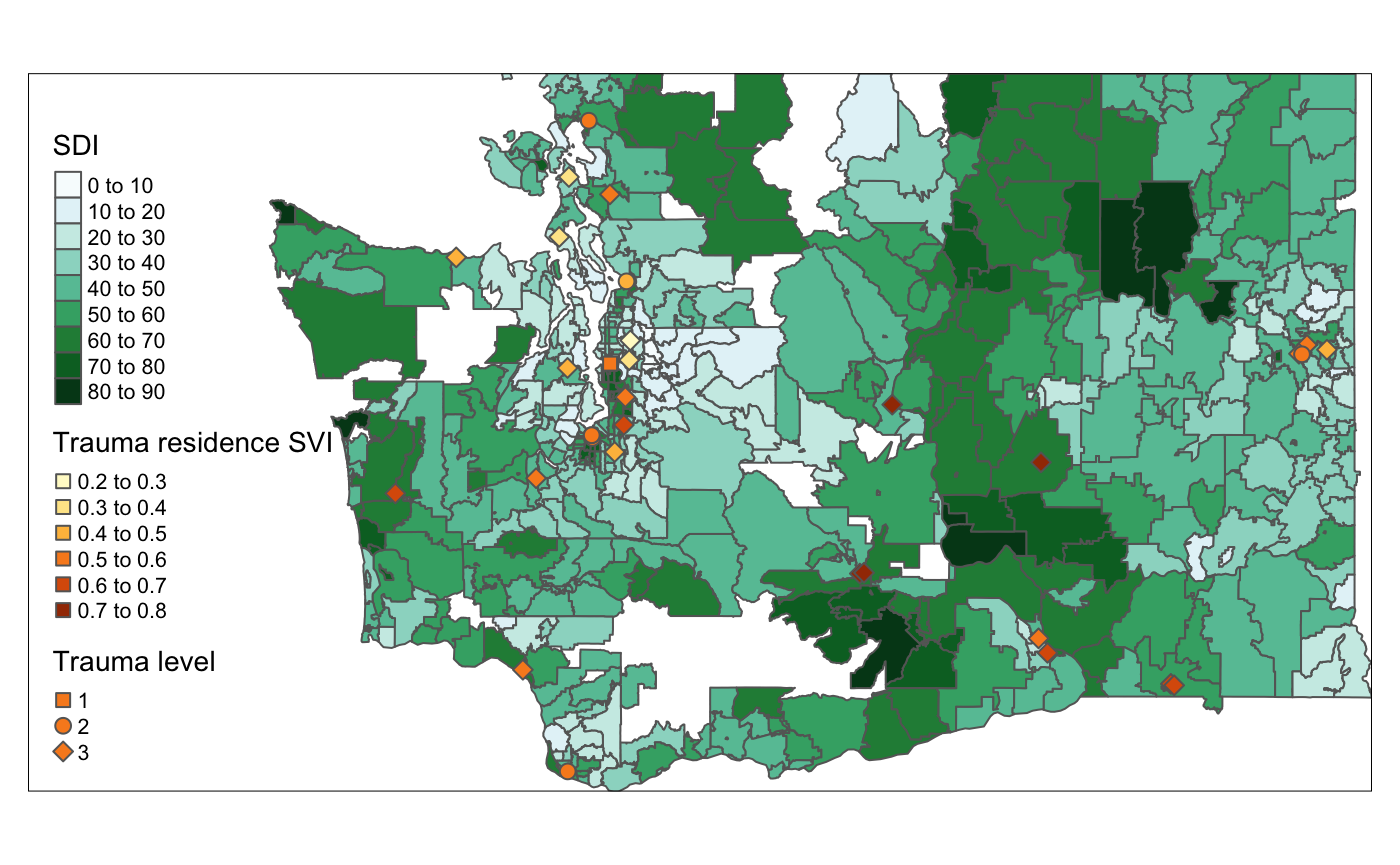


Each symbol represents a TC/non-TC.

Average patient SVI: calculated as the average SVI among all the patient residence zip codes for each TC/non-TC.

SA.2-2 Fig: SDI of hospital zip code with average patient SVI in level I, II, III TCs in WA state

SA-3 Table: Clustering features of set 1 and set 2

| **Feature category** | **Feature description** | **# Features** |
| --- | --- | --- |
|  |  |  |
| Gender | % Male in trauma admissions | 1 |
|  | % Male in non-trauma admissions | 1 |
| Age | Age median in trauma admissions | 1 |
|  | Age median in non-trauma admissions | 1 |
| Admission type | # Trauma admission | 1 |
|  | % Trauma admission in total admissions | 1 |
| Transfer type | % Transfer-in in trauma admissions | 1 |
|  | % Transfer-out in trauma admissions | 1 |
|  | % Transfer-in in non-trauma admissions | 1 |
|  | % Transfer-out in non-trauma admissions | 1 |
| Insurance payer type | % Private payer in trauma admissions | 1 |
|  | % Private payer in non-trauma admissions | 1 |
|  | % low-income payer in trauma admissions | 1 |
|  | % low-income payer in non-trauma admissions | 1 |
| ISS | Min ISS median in trauma admissions | 1 |
|  | % Min ISS over 15 in trauma admissions (out of # of min ISS exists) | 1 |
| Mechanism type | % Blunt in trauma admissions | 1 |
|  | % Penetrating in trauma admissions | 1 |
|  | % Burn in trauma admissions | 1 |
| Social Index | SDI in the TC/non-TC area | 1 |
|  | SVI mean in trauma admissions’ residence | 1 |
|  | SVI mean in non-trauma admissions’ residence | 1 |
| MPs | # MPs carried out for non-trauma admissions | 1 |
|  | # MPs carried out for trauma admissions | 1 |
|  | % All the 6 subgroups in total MPs for non-trauma admissions | 6 |
|  | % All the 6 subgroups in total MPs for trauma admissions | 6 |
| PCG volume | Cluster labels of # PCG in Major General Surgery for trauma admissions | 1 |
|  | Cluster labels of # PCG in Major Orthopedics for trauma admissions | 1 |
|  | Cluster labels of # PCG in Major Neurosurgery for trauma admissions | 1 |
|  | Cluster labels of # PCG in Major Urology for trauma admissions | 1 |
|  | Cluster labels of # PCG in Major Subspecialty for trauma admissions | 1 |
| % PCG | % Each PCG in MPs for trauma admissions | 438 |

Abbreviations: ISS (Injury Severity Score); SDI (Social Deprivation Index); SVI (Social Vulnerability Index); MP (major therapeutic procedures); PCG (Procedure Complexity Group).

**SA.3. Clustering analysis method supplemental information**

For Set 1 and Set 2, we standardized the original features to be on the same scale. Specifically, we standardized the features that are not in the [0, 1] range to [0, 1] using the min-max standardization [6]:

$$z_{i}=\frac{x_{i}-min(x)}{\max\left( x \right)-min(x)}$$

where min(x)/max(x) is the minimum/maximum value of this feature among all hospitals. For SDI with a range of [0, 100], we divided it by 100 to scale it down to the [0, 1] range. For Set 3-1 and Set 3-2, the features are already on the same scale, therefore, no standardization is needed. For each set, we carried out Principal Component Analysis (PCA) on the standardized features to reduce the dimensions of the original features and remove collinearity. PCA is an orthogonal linear transformation of the original features into new independent features denoted as principal components [7]. The 1^st^ principal component is the most important since it contains the most variation in the data, the 2^nd^ principal component contains the second most variation, and so on [7]. We selected the top components reaching 90% of the total variation [7]. We conducted an unsupervised clustering analysis using Partition Around Medoids (PAM) [8] method on WA TCs/non-TCs based on the selected principal components. We chose the number of clusters mainly based on the Silhouette method, which is a measure of how similar a TC/non-TC is to its own cluster compared to other clusters [9]. We displayed the results using t-distributed stochastic neighbor embedding (t-SNE) [10], which is a statistical method for visualizing high-dimensional data by giving each data point a location in a two or three-dimensional map.

**SB. Supplemental results**

SB-1 Table: Summary of surgical care and other features by trauma center level for all WA hospitals (TCs and non-TCs)

|  | **Trauma center level** | | | | | | |  |
| --- | --- | --- | --- | --- | --- | --- | --- | --- |
| **TC/non-TC features** | **Total** | **I** | **II** | **III** | **IV** | **V** | **Non-Trauma** | **P-value^a^** |
| Number of hospitals | 100 | 1 | 7 | 24 | 35 | 14 | 19 | / |
| **Admission type** |  |  |  |  |  |  |  |  |
| Total admissions, n | 635,973 | 14,747 | 152,800 | 242,018 | 85,695 | 4562 | 136,151 | / |
| Total trauma admission, n (%) | 34,645 (5%) | 5605 (38%) | 9011 (6%) | 11,364 (5%) | 4277 (5%) | 240 (5%) | 4148 (3%) | <0.001 |
| Trauma Admissions, per hospital, med (IQR) | 102.5 (13.75, 424.75) | 5605 | 1305 (1066, 1632) | 425 (237.5, 647.2) | 66 (19, 137.5) | 9 (2, 15.25) | 17 (3.5, 338.5) | < 0.001 |
| **Gender** |  |  |  |  |  |  |  |  |
| Admissions Female, n (%) | 362747 (57%) | 5295 (36%) | 86058 (56%) | 142417 (59%) | 49285 (58%) | 2827 (62%) | 76865 (56%) | <0.001 |
| Trauma admissions female, n (%) | 17168 (50%) | 1758 (31%) | 4253 (47%) | 6143 (54%) | 2520 (59%) | 153 (64%) | 2341 (56%) | <0.001 |
| Female trauma patients, by hospital, med (IQR) | 62 (9, 242) | 1758 | 594 (509.5, 800.5) | 240.5 (117, 353.8) | 42 (9, 76.5) | 8 (5, 10.5) | 4 (2, 186.5) | <0.001 |
| **Age** |  |  |  |  |  |  |  |  |
| Age in years- all patients, med (IQR) | 53 (27, 70) | 53 (36, 65) | 53 (25, 70) | 54 (27, 72) | 59 (32, 74) | 55 (25, 75) | 46 (22, 67) | 0.005 |
| Age in years- trauma patients, med (IQR) | 66 (46, 81) | 47 (28, 64) | 63 (41, 78) | 71 (56, 84) | 73 (58, 85) | 75 (62, 86) | 69 (49, 83) | <0.001 ^b^ |
| **Transfer type** |  |  |  |  |  |  |  |  |
| Trauma admissions transferred in, n (%) | 4897 (14%) | 2832 (51%) | 734 (8%) | 485 (4%) | 86 (2%) | 0 (0%) | 760 (18%) | <0.001 |
| Trauma admissions transferred in, per hospital, med (IQR) | 1 (0, 11)^c^ | 2832 | 65 (40, 110.5) | 7.5 (1, 23.75) | 1 (0, 2.5) | 0 (0, 0) | 3 (1, 47) | < 0.001 |
| Patients transferred out total, n (% in admission) | 15128 (2%) | 204 (1%) | 1752 (1%) | 5447 (2%) | 4426 (5%) | 492 (11%) | 2807 (2%) | <0.001 |
| Trauma patients transferred out, n (%)^d^ | 996 (3%) | 80 (1%) | 183 (2%) | 313 (3%) | 261 (6%) | 49 (20%) | 110 (3%) | <0.001 |
| Trauma patients transferred out, per hospital ^e^, med (IQR) | 6 (2, 12.25) | 80 | 18 (15.5, 31.5) | 12 (8.75, 15.75) | 6 (4, 9.5) | 2 (0, 5.75) | 1 (0, 6.5) | <0.001 |
| **Payer type in trauma admissions^f^** |  |  |  |  |  |  |  |  |
| Private, n (%) | 10286 (30%) | 2243 (40%) | 2829 (31%) | 2975 (26%) | 1122 (26%) | 49 (20%) | 1068 (26%) | <0.001 |
| Low Income, n (%) | 6394 (18%) | 1882 (34%) | 1975 (22%) | 1303 (11%) | 576 (13%) | 27 (11%) | 631 (15%) |  |
| Other, n (%)^f^ | 17965 (52%) | 1480 (26%) | 4207 (47%) | 7086 (62%) | 2579 (60%) | 164 (68%) | 2449 (59%) |  |
| Percent private per hospital, med % (IQR) | 23% (11%, 29%) | 40% | 31% (29%, 35%) | 23% (17%, 26%) | 18% (9%, 28%) | 8% (3%, 23%) | 24% (22%, 33%) | 0.01 |
| Percent low-income per hospital, med % (IQR) | 13% (6%, 19%) | 34% | 20% (17%, 27%) | 12% (9%, 14%) | 11% (3%, 17%) | 11% (3%, 16%) | 12% (2%, 42%) | 0.09 |
| Percent other per hospital, med % (IQR) | 65% (52%, 75%) | 26% | 52% (36%, 53%) | 64% (58%, 69%) | 70% (55%, 84%) | 80% (62%, 88%) | 64% (11%, 71%) | 0.007 |
| **Injury Severity Score (ISS)** |  |  |  |  |  |  |  |  |
| ISS^c^, med (IQR) | 4 (1, 10) | 9 (2, 19) | 6 (1, 16) | 4 (1, 9) | 4 (1, 9) | 4 (1, 9) | 4 (1, 9) | 0.007 ^g^ |
| **Injury Mechanism in trauma admissions** |  |  |  |  |  |  |  |  |
| Total Blunt, n (%) | 25384 (73%) | 3837 (68%) | 6761 (75%) | 8676 (76%) | 3151 (74%) | 151 (63%) | 2808 (68%) | <0.001 |
| Total Penetrating, n (%) | 1503 (4%) | 435 (8%) | 470 (5%) | 341 (3%) | 149 (3%) | *** | 107 (3%) |  |
| Total Burn, n (%) | 540 (2%) | 411 (7%) | 48 (1%) | 51 (0.4%) | 13 (0.3%) | *** | 13 (0.3%) |  |
| Total Other, n (%) | 3247 (9%) | 372 (7%) | 914 (10%) | 1125 (10%) | 429 (10%) | 14 (6%) | 393 (9%) |  |
| Total Missing, n (%) | 3971 (11%) | 550 (10%) | 818 (9%) | 1171 (10%) | 535 (13%) | 70 (29%) | 827 (20%) |  |
| Blunt per hospital, med (IQR) | 82 (8, 314) | 3837 | 973 (779, 1257.5) | 334.5 (182.2, 500.5) | 51 (14.5, 101) | 5 (4, 13) | 1 (0, 219.5) | <0.001 |
| Penetrating per hospital, med (IQR) | 3 (0, 13) | 435 | 71 (46, 90) | 12.5 (8, 19.25) | 2 (0, 5.5) | 0 (0, 0) | 0 (0, 5.5) | <0.001 |
| Burn per hospital, med (IQR) | 0 (0, 2) | 411 | 7 (5.5, 7) | 2 (1, 3.25) | 0 (0, 0.5) | 0 (0, 1) | 0 (0, 1) | <0.001 |
| Other per hospital, med (IQR) | 7 (0, 44) | 372 | 140 (103, 172) | 43 (18.75, 55.25) | 3 (1, 11.5) | 0 (0, 0) | 0 (0, 28) | <0.001 |
| **Social Index** |  |  |  |  |  |  |  |  |
| SDI in hospital area, by hospital, med (IQR) | 54 (45.6, 65.83) | 73.88 | 75 (68.73, 91.5) | 54.06 (44.73, 69.01) | 53.67 (48.31, 61.08) | 46.4 (42.33, 53.38) | 49.67 (40.72, 66.11) | 0.01 |
| SVI in trauma admissions’ residence, med (IQR) | 0.51 (0.36, 0.65) | 0.51 (0.35, 0.66) | 0.52 (0.41, 0.65) | 0.51 (0.36, 0.65) | 0.53 (0.40, 0.67) | 0.44 (0.41, 0.70) | 0.40 (0.18, 0.57) | <0.001 |
| SVI in non-trauma admissions’ residence, med (IQR) | 0.52 (0.36, 0.66) | 0.56 (0.40, 0.68) | 0.52 (0.42, 0.65) | 0.52 (0.37, 0.69) | 0.55 (0.43, 0.69) | 0.50 (0.41, 0.78) | 0.44 (0.25, 0.63) | <0.001 |
| **Major Therapeutic Procedure (MP)** |  |  |  |  |  |  |  |  |
| Total MP, n | 322878 | 16755 | 77357 | 108322 | 33534 | 1026 | 85884 | / |
| Unique MP for trauma, n | 3420 | 1688 | 1578 | 1474 | 694 | 41 | 1036 | / |
| MP for trauma, n (% in total MP) | 28418 (9%) | 8025 (48%) | 6645 (9%) | 7965 (7%) | 2578 (8%) | 79 (8%) | 3126 (4%) | <0.001 |
| MP for trauma by hospital, med (IQR) | 198 (65, 436) | 8025 | 911 (708, 1040) | 276.5 (139.8, 451.5) | 67.5 (13.75, 147.25) | 39.5 (36.25, 42.75) | 292 (198, 432) | <0.001 |
| General Surgery for trauma, n (% in MP for trauma) | 3122 (11%) | 1042 (13%) | 934 (14%) | 642 (8%) | 114 (4%) | 0 (0%) | 390 (12%) | <0.001 |
| Orthopedics for trauma, n (% in MP for trauma) | 19016 (67%) | 4291 (53%) | 4286 (64%) | 6218 (78%) | 2185 (85%) | 75 (95%) | 1961 (63%) |  |
| Neurosurgery for trauma, n (% in MP for trauma) | 3686 (13%) | 1436 (18%) | 840 (13%) | 711 (9%) | 122 (5%) | 2 (3%) | 575 (18%) |  |
| Urology for trauma, n (% in MP for trauma) | 187 (1%) | 43 (1%) | 35 (1%) | 57 (1%) | 19 (1%) | 0 (0%) | 33 (1%) |  |
| Subspecialty for trauma, n (% in MP for trauma) | 2246 (8%) | 1203 (15%) | 511 (8%) | 279 (4%) | 111 (4%) | 2 (3%) | 140 (4%) |  |
| Other MP for trauma, n (% in MP for trauma) | 167 (1%) | 10 (0.1%) | 42 (1%) | 62 (1%) | 30 (1%) | 0 (0%) | 23 (1%) |  |
| General Surgery for non-trauma, n (% in MP for non-trauma) | 108070 (37%) | 2025 (23%) | 31943 (45%) | 33737 (34%) | 8860 (29%) | 164 (17%) | 31341 (38%) | <0.001 |
| Orthopedics for non-trauma, n (% in MP for non-trauma) | 58887 (20%) | 2048 (23%) | 11783 (17%) | 20860 (21%) | 9320 (30%) | 263 (28%) | 14613 (18%) |  |
| Neurosurgery for non-trauma, n (% in MP for non-trauma) | 40083 (14%) | 3274 (38%) | 7819 (11%) | 13234 (13%) | 3947 (13%) | 7 (1%) | 11802 (14%) |  |
| Urology for non-trauma, n (% in MP for non-trauma) | 13206 (4%) | 273 (3%) | 2768 (4%) | 4060 (4%) | 1296 (4%) | 48 (5%) | 4761 (6%) |  |
| Subspecialty for non-trauma, n (% in MP for non-trauma) | 12365 (4%) | 968 (11%) | 2521 (4%) | 2374 (2%) | 824 (3%) | 11 (1%) | 5667 (7%) |  |
| Other MP for non-trauma, n (% in MP for non-trauma) | 61767 (21%) | 98 (1%) | 14015 (20%) | 26149 (26%) | 6697 (22%) | 451 (48%) | 14357 (17%) |  |

a: Statistical tests were applied based on the distribution of the data point. Obtain p-values for medians from Krusal-Wallis test, and for proportions from Chi-squared test.

b. p-value 0.2724 for level 1 and 2; <0.001 for level 3, 4, 5, and non-trauma.

c. Mean transfers in 46, skewed data.

d. Does not include patients who transferred from the emergency department without hospital admission.

e. Trauma patients only, minimum value, as some diagnostic codes did not convert.

f. Payer type: private (health maintenance organization, commercial insurance, labor and industries, or health care service contractor), low-income payer (Medicaid, self-pay, or charity care) or other payer which includes Medicare.

g. p-value 0.3491 for level 1 and 2; 0.1457 for level 3, 4, 5, and non-trauma.

*** categories with <10 admissions in the raw count are not reported per the data use agreement.

SB-2 Table: Subgroup clustering for set 1

| **Cluster labels of** | **# Features** | **Optimal cluster number in [3, 10] by the Silhouette method** |
| --- | --- | --- |
| # PCG in Major General Surgery for trauma admissions | 128 | 3 |
| # PCG in Major Orthopedics for trauma admissions | 139 | 3 |
| # PCG in Major Neurosurgery for trauma admissions | 77 | 3 |
| # PCG in Major Urology for trauma admissions | 12 | 5 |
| # PCG in Major Subspecialty for trauma admissions | 83 | 3 |


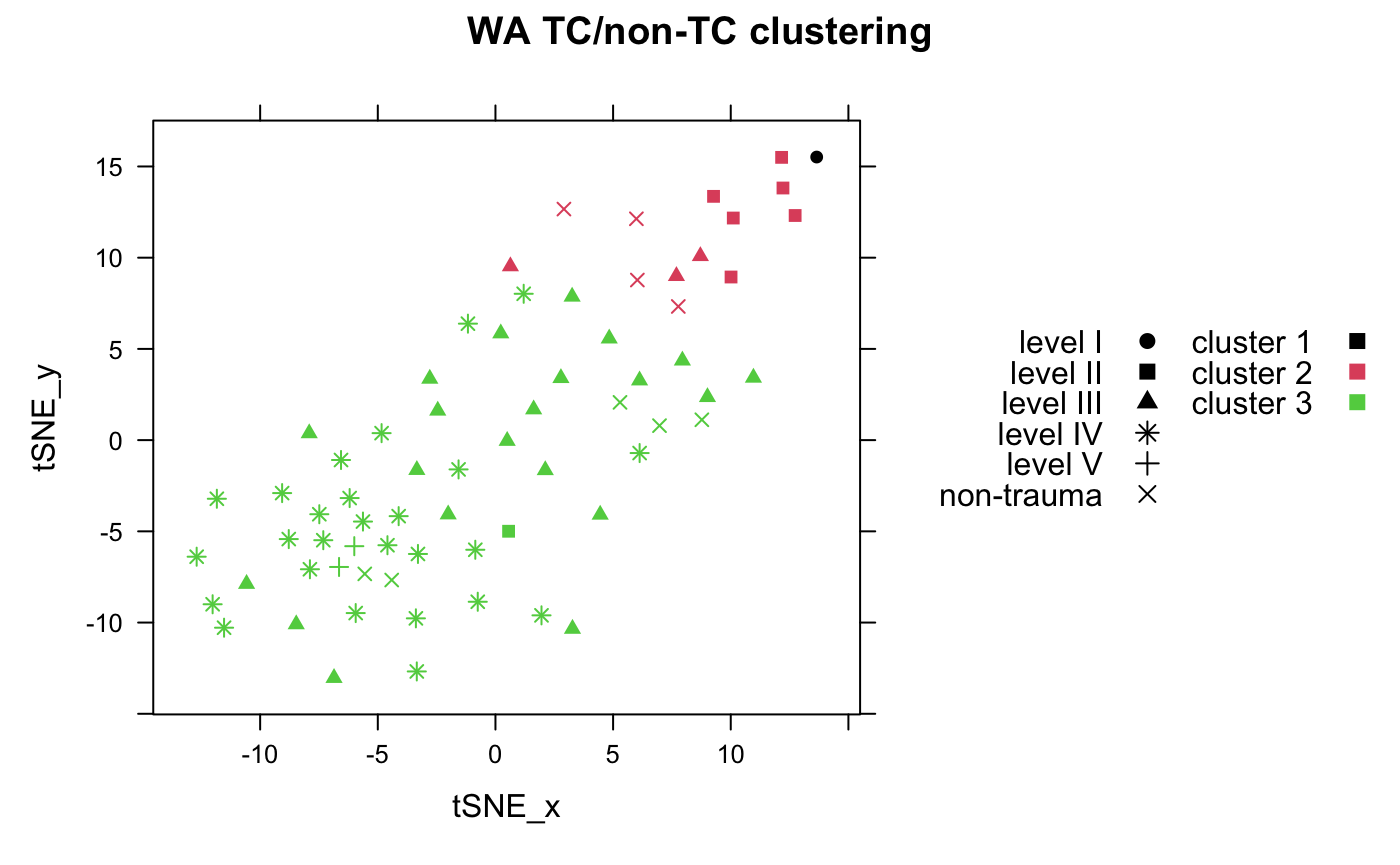


SB-1 Fig: Subgroup clustering results for general surgery


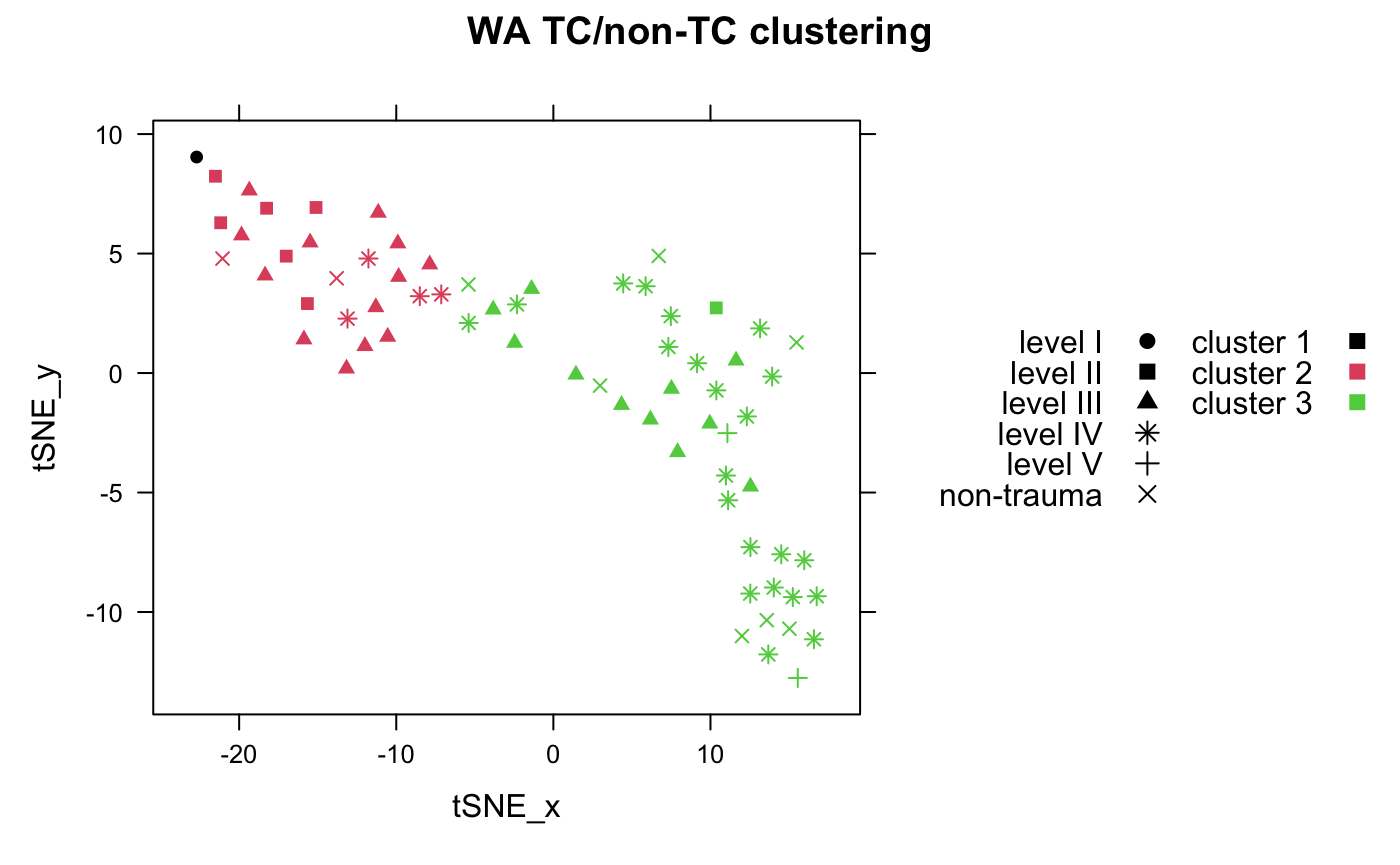


SB-2 Fig: Subgroup clustering results for orthopedics


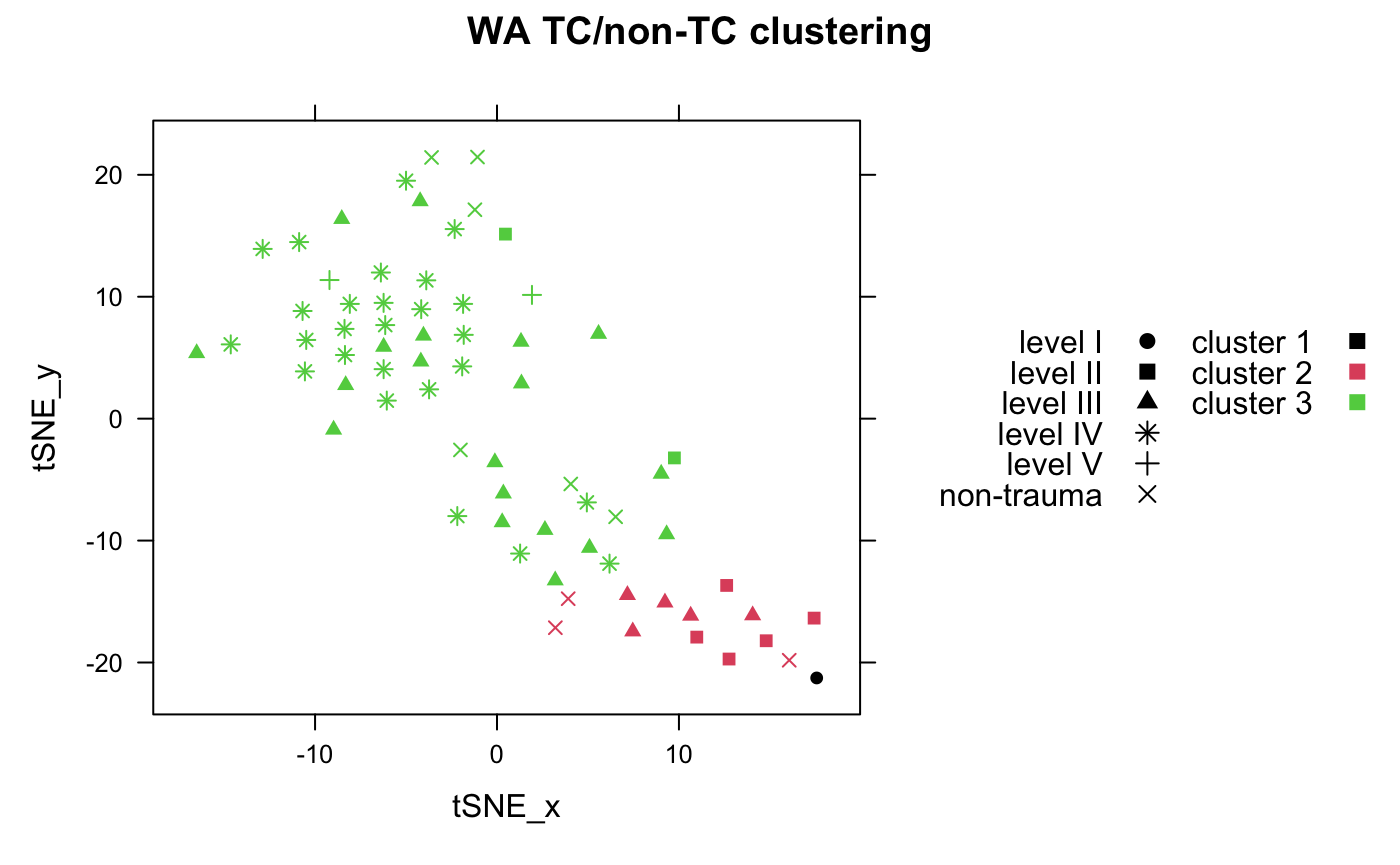


SB-3 Fig: subgroup clustering results for neurosurgery


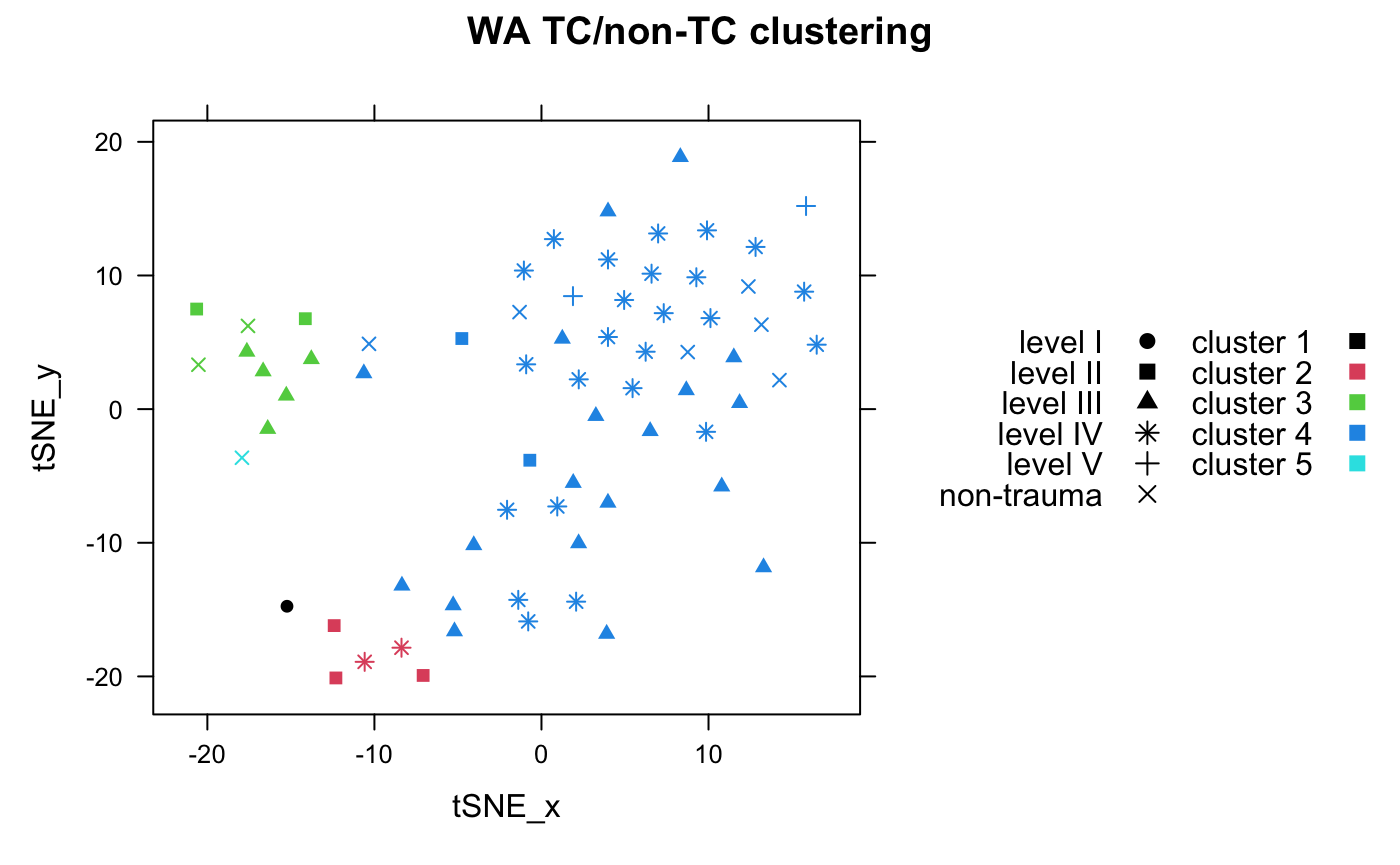


SB-4 Fig: subgroup clustering results for urology


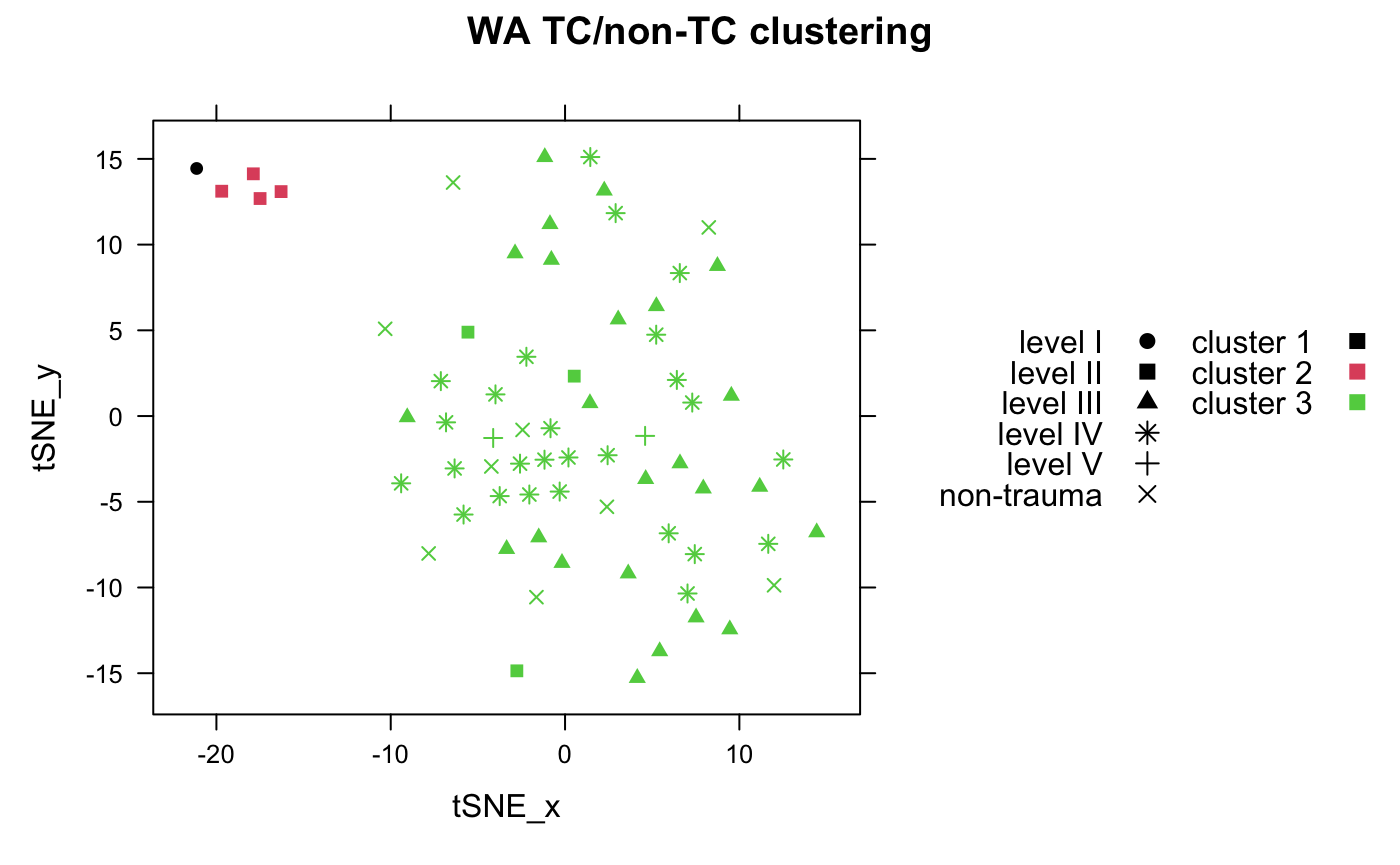


SB-5 Fig: Subgroup clustering results for subspecialty


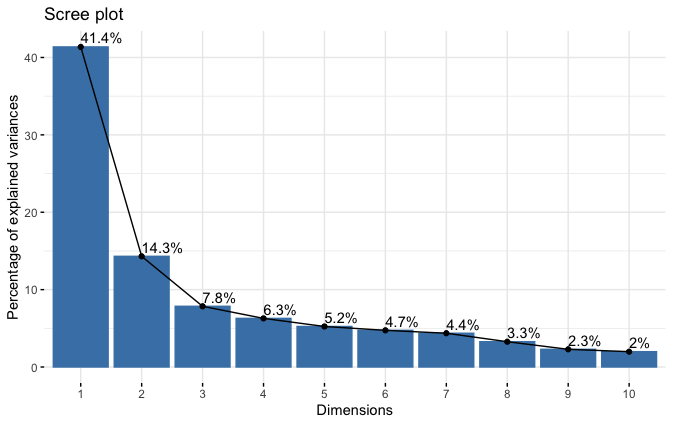


SB-6 Fig: PCA result of set 1


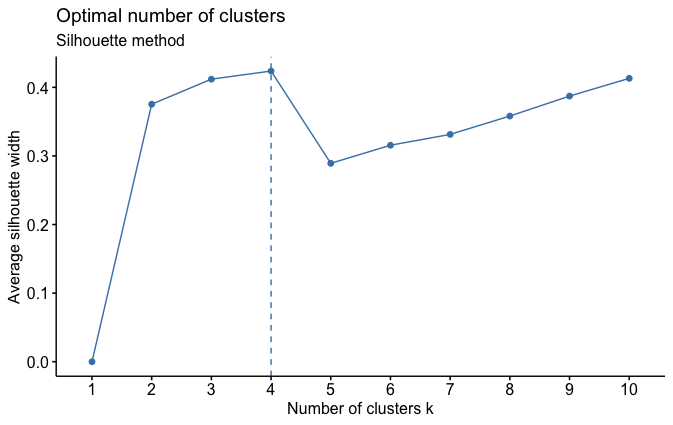


SB-7 Fig: Cluster number evaluation of set 1


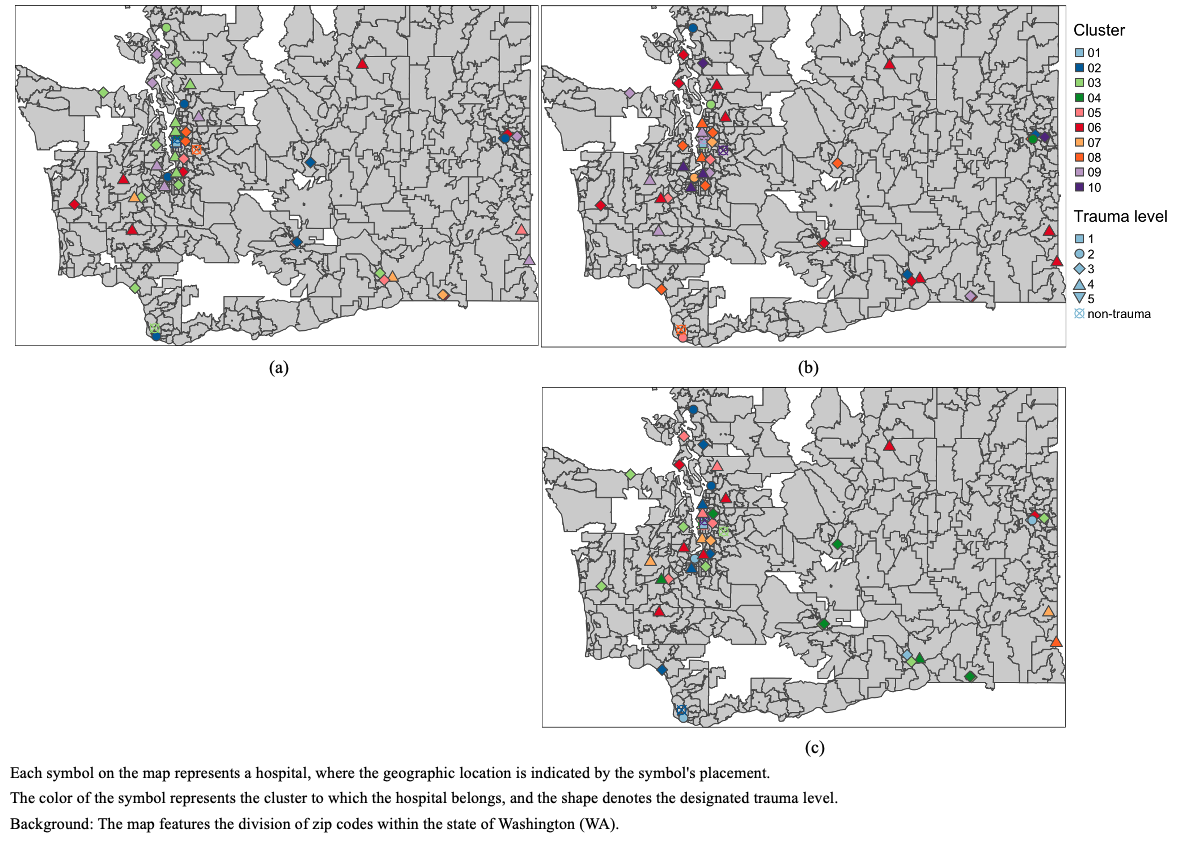


The background of the map illustrates the division of zip codes within the state of Washington (WA).

Each symbol on the map represents a hospital, where the geographic location is indicated by the symbol's placement.

The color of the symbol represents the cluster to which the hospital belongs, and the shape denotes the designated trauma level.

SB-8 Fig: Clustering results displayed on a map of WA:

(a) Set 2 surgical care PCG distribution and other features clustering,

(b) Set 3-1 surgical care volume clustering,

(c) Set 3-2 surgical care distribution clustering

SB-3 Table: Original features contributed to the TCs/non-TCs clusters from Set 1 surgical care procedure subgroup labels and other features clustering

| **Cluster** | 1 | 2 | 3 | 4 | 5 | 6 | 7 | 8 | 9 | 10 |
| --- | --- | --- | --- | --- | --- | --- | --- | --- | --- | --- |
| **TC/non-TC levels in the cluster** | Ⅰ | Ⅱ | Ⅱ, Ⅲ,  Non | Ⅱ, Ⅲ | Ⅱ, Non | Ⅲ, Ⅳ,  Non | Ⅲ, Ⅳ,  Ⅴ, Non | Ⅲ, Non | Ⅲ, Ⅳ | Ⅲ |
| **# TC/non-TC in the cluster** | 1 | 3 | 5 | 3 | 3 | 12 | 26 | 4 | 10 | 2 |
| **Cluster mean** |  | | | | | | | | | |
| Median age of trauma patients (year) | 47 | 61 | 70 | 72 | 7 | 70 | 72 | 70 | 70 | 74 |
| Median age of non-trauma patients (year) | 55 | 50 | 47 | 57 | 8 | 54 | 56 | 61 | 34 | 57 |
| # Trauma admissions | 5605 | 1552 | 1345 | 688 | 182 | 601 | 173 | 378 | 37 | 545 |
| % Trauma patients transferred out | 1% | 2% | 1% | 2% | 2% | 3% | 6% | 3% | 27% | 3% |
| Median ISS | 9 | 9 | 4 | 4 | 3 | 4 | 4 | 7 | 4 | 4 |
| SDI in TC/non-TC area | 74 | 87 | 62 | 70 | 63 | 52 | 50 | 65 | 64 | 45 |
| Mean SVI in trauma patient residence | 0.51 | 0.56 | 0.39 | 0.49 | 0.55 | 0.56 | 0.52 | 0.47 | 0.78 | 0.45 |
| Mean SVI in non-trauma patient residence | 0.54 | 0.56 | 0.40 | 0.51 | 0.53 | 0.58 | 0.53 | 0.50 | 0.80 | 0.46 |
| % Trauma patients with private payer | 40% | 34% | 26% | 21% | 41% | 31% | 22% | 25% | 15% | 18% |
| % Trauma patients with low-income payer | 34% | 25% | 13% | 13% | 56% | 12% | 12% | 12% | 23% | 10% |
| % Non-trauma patients with private payer | 22% | 35% | 46% | 30% | 43% | 39% | 34% | 35% | 23% | 30% |
| % Non-trauma patients with low-income payer | 39% | 31% | 19% | 25% | 54% | 22% | 22% | 15% | 48% | 25% |
| # Trauma MP | 8025 | 1296 | 923 | 452 | 112 | 444 | 103 | 325 | 19 | 349 |
| # Non-trauma MP | 8730 | 13474 | 14318 | 6736 | 3113 | 4759 | 1406 | 10030 | 275 | 3602 |
| % Trauma major General Surgery in all MP | 13% | 16% | 10% | 13% | 7% | 6% | 5% | 20% | 11% | 5% |
| % Trauma major Orthopedics in all MP | 53% | 58% | 73% | 74% | 67% | 83% | 85% | 40% | 73% | 85% |
| % Trauma major Neurosurgery in all MP | 18% | 16% | 10% | 9% | 19% | 6% | 6% | 34% | 0% | 4% |
| % Non-trauma major General Surgery in all MP | 23% | 45% | 36% | 48% | 33% | 30% | 31% | 40% | 17% | 27% |
| % Non-trauma major Orthopedics in all MP | 23% | 15% | 20% | 16% | 30% | 29% | 28% | 12% | 14% | 35% |
| % Non-trauma major Neurosurgery in all MP | 38% | 13% | 9% | 9% | 22% | 10% | 8% | 29% | 2% | 5% |
| % Non-trauma other major procedures in all MP | 1% | 20% | 29% | 19% | 0% | 23% | 24% | 9% | 59% | 25% |
| % TC/non-TC in General Surgery label 1 | 100%^*^ | 0 | 0 | 0 | 0 | 0 | 0 | 0 | 0 | 0 |
| % TC/non-TC in General Surgery label 2^a7^ | 0 | 100% | 80% | 100% | 0 | 0 | 0 | 75% | 0 | 0 |
| % TC/non-TC in General Surgery label 3^a4^ | 0 | 0 | 20% | 0 | 100% | 100% | 100% | 25% | 100% | 100% |
| % TC/non-TC in Orthopedics label 1 | 100% | 0 | 0 | 0 | 0 | 0 | 0 | 0 | 0 | 0 |
| % TC/non-TC in Orthopedics label 2^a3, b1^ | 0 | 100% | 100% | 100% | 0 | 100% | 0 | 0 | 0 | 100% |
| % TC/non-TC in Orthopedics label 3^a1, b2^ | 0 | 0 | 0 | 0 | 100% | 0 | 100% | 100% | 100% | 0 |
| % TC/non-TC in Neurosurgery label 1 | 100% | 0 | 0 | 0 | 0 | 0 | 0 | 0 | 0 | 0 |
| % TC/non-TC in Neurosurgery label 2^a6^ | 0 | 100% | 100% | 0 | 0 | 17% | 0 | 75% | 0 | 0 |
| % TC/non-TC in Neurosurgery label 3^a2^ | 0 | 0 | 0 | 100% | 100% | 83% | 100% | 25% | 100% | 100% |
| % TC/non-TC in Urology label 1 | 100% | 0 | 0 | 0 | 0 | 0 | 0 | 0 | 0 | 0 |
| % TC/non-TC in Urology label 2 | 0 | 100% | 0 | 0 | 0 | 0 | 8% | 0 | 0 | 0 |
| % TC/non-TC in Urology label 3^c1^ | 0 | 0 | 80% | 0 | 0 | 0 | 0 | 75% | 0 | 100% |
| % TC/non-TC in Urology label 4^a5^ | 0 | 0 | 0 | 100% | 100% | 100% | 92% | 25% | 100% | 0 |
| % TC/non-TC in Urology label 5 | 0 | 0 | 20% | 0 | 0 | 0 | 0 | 0 | 0 | 0 |
| % TC/non-TC in Subspecialty label 1 | 100% | 0 | 0 | 0 | 0 | 0 | 0 | 0 | 0 | 0 |
| % TC/non-TC in Subspecialty label 2 | 0 | 100% | 20% | 0 | 0 | 0 | 0 | 0 | 0 | 0 |
| % TC/non-TC in Subspecialty label 3 | 0 | 0 | 80% | 100% | 100% | 100% | 100% | 100% | 100% | 100% |

ai (i = 1, 2, …, 7): The top ith feature that contributes no less than 10% of the variation within the 1^st^ principal component

bi (i = 1, 2): The top ith feature that contributes no less than 10% of the variation within the 2^nd^ principal component

c1: The 1^st^ and only feature that contributes no less than 10% of the variation within the 3^rd^ principal component

*: 100% percent of the TCs/non-TCs in cluster 1 are with general surgery label 1

Abbreviations: ISS (Injury Severity Score); TC (Trauma Center); SDI (Social Deprivation Index); SVI (Social Vulnerability Index); MP (major therapeutic procedures).


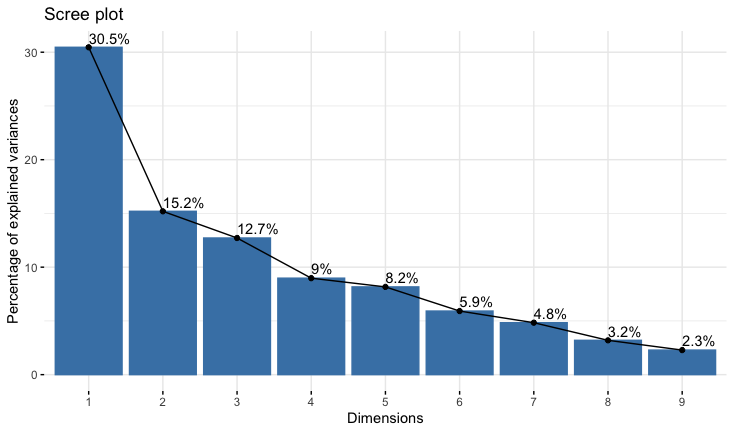


SB-9 Fig: PCA result of set 2


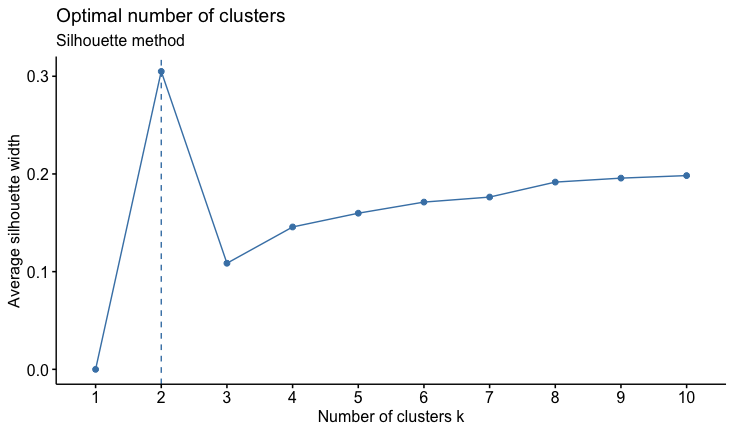


SB-10 Fig: Cluster number evaluation of set 2

SB-4 Table: Original features contributed to the TCs/non-TCs clusters from Set 2 surgical care PCG distribution and other features clustering

| **Cluster** | 1 | 2 | 3 | 4 | 5 | 6 | 7 | 8 | 9 | 10 |
| --- | --- | --- | --- | --- | --- | --- | --- | --- | --- | --- |
| **TC/non-TC levels in the cluster** | Ⅰ | Ⅱ, Ⅲ,  Non | Ⅱ, Ⅲ,  Ⅳ, Non | Ⅱ, Non | Ⅲ, Ⅳ | Ⅲ, Ⅳ | Ⅲ, Ⅳ | Ⅲ, Non | Ⅲ, Ⅳ | Non |
| **# TC/non-TC in the cluster** | 1 | 11 | 14 | 2 | 4 | 7 | 3 | 3 | 7 | 1 |
| **Cluster mean** |  |  |  |  |  |  |  |  |  |  |
| % Trauma patients who were male | 69% | 51% | 44% | 57% | 46% | 43% | 50% | 42% | 43% | 57% |
| % Non-trauma who were male | 61% | 45% | 42% | 54% | 38% | 39% | 44% | 38% | 45% | 53% |
| Median age of trauma patients (year)^b2^ | 47 | 65 | 72 | 9 | 70 | 71 | 69 | 75 | 74 | 72 |
| Median age of non-trauma patients (year)^b1, c2^ | 55 | 54 | 56 | 6 | 38 | 52 | 60 | 43 | 62 | 66 |
| # Trauma admissions^d3^ | 5605 | 1042 | 547 | 270 | 434 | 225 | 188 | 784 | 235 | 495 |
| % Trauma admissions | 38% | 5% | 5% | 4% | 4% | 6% | 5% | 5% | 7% | 5% |
| % Trauma patients transferred in | 51% | 10% | 3% | 16% | 1% | 2% | 6% | 6% | 1% | 46% |
| % Trauma patients transferred out | 1% | 2% | 3% | 3% | 3% | 7% | 3% | 2% | 5% | 3% |
| % Non-trauma patients transferred in | 18% | 8% | 2% | 11% | 1% | 1% | 3% | 5% | 1% | 23% |
| Median ISS | 9 | 6 | 4 | 5 | 4 | 4 | 4 | 4 | 4 | 16 |
| % Median ISS over 15 (severe injured) | 40% | 22% | 11% | 19% | 11% | 8% | 13% | 11% | 6% | 55% |
| % Trauma patients with a blunt mechanism | 68% | 71% | 75% | 57% | 73% | 79% | 74% | 75% | 76% | 75% |
| % Trauma patients with a penetrating mechanism | 8% | 4% | 4% | 11% | 3% | 2% | 3% | 2% | 4% | 0% |
| % Trauma patients with a burn mechanism | 7% | 0% | 0% | 1% | 0% | 0% | 0% | 0% | 1% | 0% |
| SDI in TC/non-TC area^c3, d2^ | 74 | 82 | 46 | 62 | 63 | 66 | 56 | 24 | 41 | 61 |
| Mean SVI in trauma patient residence^c4^ | 0.51 | 0.53 | 0.50 | 0.53 | 0.59 | 0.66 | 0.63 | 0.30 | 0.43 | 0.40 |
| Mean SVI in non-trauma patient residence^c5^ | 0.54 | 0.55 | 0.51 | 0.52 | 0.60 | 0.66 | 0.62 | 0.30 | 0.45 | 0.44 |
| % Trauma patients with private payer | 40% | 27% | 27% | 45% | 40% | 17% | 32% | 24% | 22% | 23% |
| % Trauma patients with low-income payer | 34% | 20% | 12% | 50% | 5% | 16% | 13% | 9% | 11% | 12% |
| % Non-trauma patients with private payer^c1^ | 22% | 34% | 35% | 42% | 61% | 20% | 40% | 56% | 28% | 31% |
| % Non-trauma patients with low-income payer | 39% | 26% | 24% | 54% | 11% | 39% | 14% | 13% | 20% | 13% |
| # Trauma MP | 8025 | 792 | 374 | 166 | 319 | 154 | 162 | 516 | 149 | 436 |
| # Non-trauma MP^d1^ | 8730 | 11912 | 4401 | 4438 | 3997 | 1279 | 3130 | 8309 | 1310 | 11040 |
| % Trauma major General Surgery in all MP | 13% | 16% | 8% | 11% | 7% | 4% | 9% | 7% | 3% | 19% |
| % Trauma major Orthopedics in all MP^a1^ | 53% | 62% | 81% | 62% | 84% | 92% | 65% | 77% | 90% | 2% |
| % Trauma major Neurosurgery in all MP | 18% | 14% | 5% | 15% | 4% | 0.4% | 22% | 10% | 3% | 76% |
| % Trauma major Subspecialty in all MP | 15% | 7% | 5% | 11% | 3% | 3% | 2% | 3% | 3% | 3% |
| % Non-trauma major General Surgery in all MP | 23% | 47% | 38% | 49% | 22% | 27% | 13% | 28% | 24% | 44% |
| % Non-trauma major Orthopedics in all MP | 23% | 17% | 22% | 15% | 22% | 27% | 31% | 18% | 47% | 3% |
| % Non-trauma major Neurosurgery in all MP | 38% | 12% | 7% | 15% | 10% | 2% | 41% | 12% | 8% | 49% |
| % Non-trauma major Urology in all MP | 3% | 5% | 5% | 5% | 6% | 5% | 2% | 4% | 5% | 0 |
| % Non-trauma major Subspecialty in all MP | 11% | 4% | 3% | 16% | 2% | 3% | 1% | 2% | 3% | 4% |
| % Non-trauma other major procedures in all MP | 1% | 16% | 25% | 0.3% | 38% | 35% | 11% | 35% | 13% | 0 |
| Percent of specific PCG in MP in trauma patients | | | | | | | | | | |
| Joint, Extremities lower, 1 | 1% | 2% | 3% | 4% | 4% | 5% | 4% | 5% | 4% | 0 |
| Joint, Extremities lower, 2 | 1% | 6% | 9% | 0 | 5% | 11% | 6% | 8% | 12% | 0 |
| Joint, Extremities lower, 3 | 0.3% | 2% | 4% | 0.3% | 2% | 5% | 3% | 4% | 5% | 0 |
| Joint, Extremities upper, 1 | 0.4% | 2% | 1% | 0 | 1% | 1% | 1% | 1% | 2% | 0 |
| Open fixation, Extremities lower, 1 | 10% | 11% | 16% | 11% | 22% | 20% | 6% | 12% | 18% | 0 |
| Open fixation, Extremities lower, 2 | 3% | 6% | 9% | 7% | 9% | 12% | 8% | 8% | 8% | 0 |
| Open fixation, Extremities lower, 3 | 5% | 7% | 8% | 4% | 9% | 10% | 7% | 8% | 10% | 0 |
| Other general surgery, Abdomen, 0 | 0.3% | 1% | 1% | 1% | 1% | 0.3% | 1% | 1% | 1% | 0.2% |
| Percutaneous fixation, Extremities lower, 2 | 1% | 3% | 5% | 2% | 6% | 7% | 2% | 6% | 7% | 0 |
| Spine procedures, Abdomen, 1 | 1% | 2% | 1% | 0.3% | 0.3% | 0 | 5% | 2% | 1% | 18% |

a1: The 1^st^ and only feature that contributes no less than 10% of the variation within the 1^st^ principal component

bi (i = 1, 2): The top ith feature that contributes no less than 10% of the variation within the 2^nd^ principal component

ci (i = 1, 2, 3, 4, 5): The top ith feature that contributes no less than 10% of the variation within the 3^rd^ principal component

di (i = 1, 2, 3): The top ith feature that contributes no less than 10% of the variation within the 4^th^ principal component

Abbreviations: ISS (Injury Severity Score); TC (Trauma Center); SDI (Social Deprivation Index); SVI (Social Vulnerability Index); MP (major therapeutic procedures); PCG (Procedure Complexity Group).


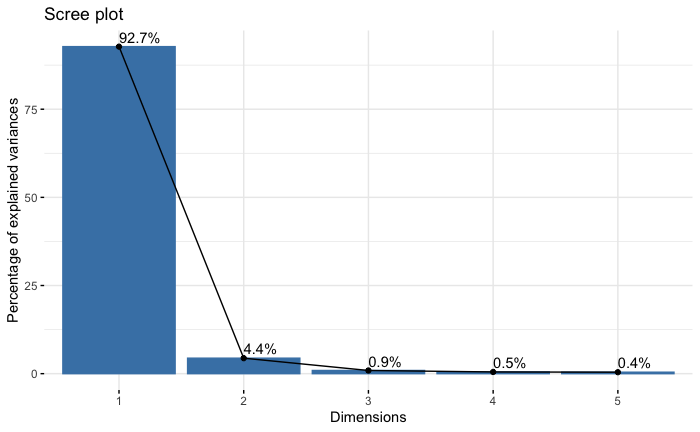


SB-11 Fig: PCA result of set 3-1


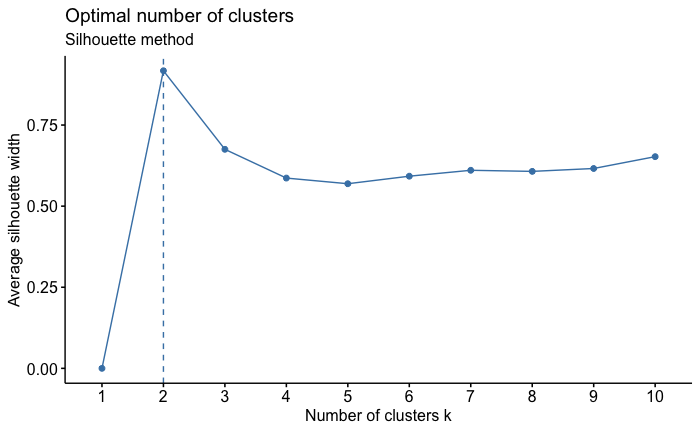


SB-12 Fig: Cluster number evaluation of set 3-1

SB-5 Table: Original features contributed to the TCs/non-TCs clusters from set 3-1 surgical care volume clustering

| **Cluster** | 1 | 2 | 3 | 4 | 5 | 6 | 7 | 8 | 9 | 10 |
| --- | --- | --- | --- | --- | --- | --- | --- | --- | --- | --- |
| **TC/non-TC levels in the cluster** | Ⅰ | Ⅱ, Ⅲ | Ⅱ, Non | Ⅱ | Ⅱ, Ⅲ | Ⅱ, Ⅲ,  Ⅳ | Ⅱ, Ⅲ | Ⅲ, Ⅳ,  Non | Ⅲ, Ⅳ,  Non | Ⅲ, Ⅳ,  Non |
| **# TC/non-TC in the cluster** | 1 | 4 | 2 | 1 | 3 | 14 | 2 | 10 | 10 | 6 |
| **Cluster mean** | | | | | | | | | | |
| Number of specific PCG in trauma patients | | | | | | | | | | |
| Amputation, Extremities upper, minor | 140 | 2 | 2 | 4 | 1 | 0 | 3 | 1 | 0 | 0.3 |
| Craniotomy, Head, major | 200 | 15 | 7 | 68 | 17 | 1 | 28 | 3 | 5 | 0 |
| Ex fix, Extremities lower, minor | 189 | 9 | 5 | 16 | 8 | 0.1 | 5 | 4 | 0.3 | 3 |
| Facial fractures, Face, minor | 145 | 7 | 6 | 45 | 9 | 0.2 | 18 | 1 | 1 | 0 |
| Joint, Extremities lower, minor | 137 | 50 | 159 | 121 | 83 | 13 | 96 | 49 | 21 | 35 |
| Open fixation, Extremities lower, major^a2^ | 511 | 51 | 103 | 185 | 91 | 8 | 66 | 37 | 13 | 24 |
| Open fixation, Extremities lower, minor^a1^ | 1069 | 131 | 252 | 390 | 224 | 20 | 166 | 95 | 36 | 65 |
| Open fixation, Extremities upper, major | 235 | 13 | 26 | 77 | 16 | 3 | 19 | 11 | 5 | 3 |
| Open fixation, Extremities upper, minor | 444 | 39 | 72 | 136 | 42 | 4 | 31 | 17 | 9 | 13 |
| Open pelvis fixation, Extremities lower, minor | 224 | 5 | 6 | 74 | 6 | 1 | 7 | 1 | 2 | 0.2 |
| Other general surgery, Abdomen, major | 166 | 11 | 11 | 32 | 12 | 2 | 19 | 2 | 3 | 1 |
| Other ortho, Extremities lower, minor | 164 | 14 | 34 | 38 | 25 | 2 | 14 | 10 | 4 | 5 |
| Other ortho, Extremities upper, minor | 232 | 12 | 28 | 29 | 14 | 2 | 22 | 7 | 2 | 3 |
| Percutaneous fixation, Extremities lower, minor | 156 | 32 | 32 | 93 | 29 | 7 | 39 | 33 | 10 | 19 |
| Percutaneous pelvic fixation, Extremities lower, minor | 146 | 1 | 0 | 23 | 2 | 0 | 0 | 0.1 | 0.1 | 0 |
| Peripheral nerve, Extremities upper, minor | 142 | 3 | 5 | 14 | 5 | 0.1 | 8 | 3 | 1 | 2 |
| Reconstruction, Extremities lower, minor | 234 | 7 | 13 | 20 | 14 | 2 | 5 | 5 | 3 | 4 |
| Reconstruction, Extremities upper, minor | 207 | 4 | 3 | 16 | 5 | 1 | 4 | 3 | 1 | 0.3 |
| Spine procedures, Abdomen, minor | 221 | 13 | 26 | 48 | 25 | 3 | 22 | 14 | 17 | 1 |
| Spine procedures, Neck, major | 318 | 12 | 13 | 37 | 12 | 1 | 29 | 4 | 12 | 0 |

ai (i = 1, 2): The top ith feature that contributes no less than 10% of the variation within the 1^st^ principal component


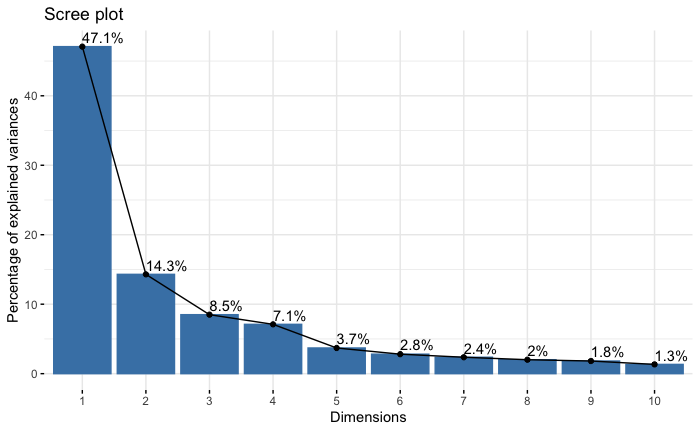


SB-13 Fig: PCA result of set 3-2

SB-6 Table: Original features contributed to the TCs/non-TCs clusters from set 3-2 surgical care distribution clustering

| **Cluster** | 1 | 2 | 3 | 4 | 5 | 6 | 7 | 8 | 9 | 10 |
| --- | --- | --- | --- | --- | --- | --- | --- | --- | --- | --- |
| **TC/non-TC levels in the cluster** | Ⅰ, Ⅱ,  Ⅲ, Non | Ⅱ, Ⅲ,  Ⅳ, Non | Ⅲ,  Non | Ⅲ, Ⅳ,  Non | Ⅲ, Ⅳ,  Non | Ⅲ,  Ⅳ | Ⅲ,  Ⅳ | Ⅳ | Non | Non |
| **# TC/non-TC in the cluster** | 9 | 8 | 9 | 7 | 6 | 7 | 4 | 1 | 1 | 1 |
| **Cluster mean** | | | | | | | | | | |
| Percent of specific PCG in MP in trauma patients | | | | | | | | | | |
| Amputation, Extremities lower, minor | 0.4% | 0.3% | 1% | 0.3% | 1% | 0.4% | 1% | 0 | 0.2% | 0 |
| Cardiac, Chest, major | 1% | 0.3% | 0.3% | 0.4% | 0.5% | 0 | 1% | 0 | 3% | 4% |
| Cardiac, Chest, minor | 1% | 1% | 1% | 1% | 1% | 0.1% | 0.3% | 0 | 6% | 8% |
| Control of hemorrhage, Abdomen, major | 1% | 0.4% | 0.3% | 2% | 0.3% | 0.1% | 0.3% | 0 | 0.2% | 0.3% |
| Craniotomy, Head, major | 3% | 1% | 0 | 1% | 1% | 0 | 1% | 0 | 8% | 1% |
| Craniotomy, Head, minor | 1% | 0.1% | 0 | 0.5% | 0.02% | 0 | 0.04% | 0 | 1% | 1% |
| Ex fix, Extremities lower, minor | 1% | 1% | 0.5% | 1% | 1% | 1% | 1% | 0 | 0 | 0 |
| Exploratory laparotomy/other abdominal  surgery, Abdomen, major | 1% | 0.3% | 0.4% | 1% | 0.1% | 0 | 0.1% | 0 | 0 | 0 |
| Joint, Extremities lower, major | 1% | 4% | 3% | 3% | 4% | 6% | 2% | 11% | 0 | 3% |
| Joint, Extremities lower, minor^b1^ | 5% | 14% | 15% | 10% | 16% | 16% | 10% | 25% | 0 | 4% |
| Joint, Extremities upper, major | 1% | 1% | 1% | 1% | 1% | 1% | 3% | 2% | 0 | 6% |
| Joint, Extremities upper, minor | 1% | 1% | 1% | 2% | 2% | 0.4% | 2% | 9% | 0 | 10% |
| Open fixation, Extremities lower, major | 8% | 10% | 9% | 7% | 9% | 11% | 10% | 13% | 0 | 4% |
| Open fixation, Extremities lower, minor^a1, b2^ | 18% | 27% | 23% | 15% | 21% | 33% | 40% | 15% | 0 | 5% |
| Open fixation, Extremities upper, major | 3% | 3% | 2% | 4% | 2% | 2% | 3% | 0 | 0 | 3% |
| Open fixation, Extremities upper, minor | 6% | 6% | 4% | 3% | 4% | 6% | 4% | 4% | 0 | 7% |
| Open pelvis fixation, Extremities lower, minor | 2% | 0.1% | 0.05% | 1% | 1% | 0 | 1% | 0 | 1% | 0 |
| Other ent, Head, minor | 1% | 0.1% | 0.04% | 0.2% | 0.01% | 0 | 0.04% | 0 | 1% | 2% |
| Other general surgery, Abdomen, major | 2% | 1% | 1% | 2% | 2% | 0.5% | 0.5% | 0 | 0 | 0 |
| Other general surgery, Abdomen, minor | 1% | 1% | 2% | 3% | 1% | 1% | 1% | 0 | 0.2% | 8% |
| Other neuro, Head, major | 1% | 0.1% | 0 | 0.3% | 0.3% | 0 | 0.1% | 0 | 3% | 1% |
| Other neuro, Head, minor | 1% | 0.1% | 0 | 0.2% | 0 | 0 | 0.1% | 0 | 0% | 0 |
| Other ortho, Extremities lower, major | 0.5% | 1% | 0.4% | 0.3% | 0.3% | 1% | 1% | 0 | 0 | 1% |
| Other ortho, Extremities lower, minor | 2% | 2% | 2% | 3% | 3% | 2% | 3% | 1% | 0.2% | 1% |
| Other ortho, Extremities upper, minor | 2% | 2% | 1% | 2% | 3% | 1% | 2% | 8% | 0.2% | 1% |
| Other thoracic, Chest, major | 1% | 0.2% | 1% | 0.4% | 0.4% | 0.5% | 0.1% | 0 | 0.2% | 0.3% |
| Percutaneous fixation, Extremities lower, major^b4^ | 2% | 3% | 8% | 3% | 3% | 5% | 1% | 1% | 0 | 1% |
| Percutaneous fixation, Extremities lower, minor^b3^ | 4% | 6% | 12% | 5% | 4% | 7% | 4% | 5% | 0 | 3% |
| Percutaneous fixation, Extremities upper, minor | 1% | 0.3% | 0.5% | 1% | 0.4% | 0.4% | 0 | 0 | 0 | 0.3% |
| Reconstruction, Extremities lower, minor | 1% | 2% | 2% | 2% | 3% | 1% | 1% | 2% | 0 | 1% |
| Reconstruction, Extremities upper, minor | 1% | 1% | 0.2% | 1% | 1% | 0.3% | 1% | 1% | 0 | 0.3% |
| Spine procedures, Abdomen, minor | 2% | 1% | 1% | 8% | 4% | 0.1% | 1% | 0 | 23% | 2% |
| Spine procedures, Chest, minor | 1% | 0.2% | 0.3% | 2% | 1% | 0 | 0.2% | 0 | 10% | 3% |
| Spine procedures, Neck, major | 2% | 1% | 0.2% | 3% | 1% | 0 | 1% | 0 | 17% | 3% |
| Spine procedures, Neck, minor | 1% | 0.2% | 0.1% | 1% | 1% | 0 | 0.3% | 0 | 7% | 1% |

a1: The only feature that contributes no less than 10% of the variation within the 1^st^ principal component

bi (i = 1, 2, 3, 4): The top ith feature that contributes no less than 10% of the variation within the 2^nd^ principal component

**References**

1. Clark DE, Black AW, Skavdahl DH, Hallagan LD. Open-access programs for injury categorization using ICD-9 or ICD-10. Inj Epidemiol. 2018;5(1):11. Epub 20180409. doi: 10.1186/s40621-018-0149-8. PubMed PMID: 29629480; PubMed Central PMCID: PMCPMC5890002.

2. Abbreviated Injury Scale Wikipedia2021. Available from: <https://en.wikipedia.org/wiki/Abbreviated_Injury_Scale>.

3. CDC/ATSDR SVI Data and Documentation Download ATSDR (Agency for Toxic Substances and Disease Registry)2021. Available from: <https://www.atsdr.cdc.gov/placeandhealth/svi/data_documentation_download.html>.

4. Social Deprivation Index (SDI) Robert Graham Center2022. Available from: <https://www.graham-center.org/maps-data-tools/social-deprivation-index.html>.

5. HUD USPS ZIP CODE CROSSWALK FILES Office of Policy Development and Research (PD&R). Available from: <https://www.huduser.gov/portal/datasets/usps_crosswalk.html>.

6. Mohamad IB, Usman D. Standardization and its effects on K-means clustering algorithm. Research Journal of Applied Sciences, Engineering and Technology. 2013;6(17):3299-303.

7. Ringnér M. What is principal component analysis? Nature biotechnology. 2008;26(3):303-4.

8. Reynolds AP, Richards G, Rayward-Smith VJ, editors. The application of k-medoids and pam to the clustering of rules. International Conference on Intelligent Data Engineering and Automated Learning; 2004: Springer.

9. Rousseeuw PJ. Silhouettes: a graphical aid to the interpretation and validation of cluster analysis. Journal of computational and applied mathematics. 1987;20:53-65.

10. Van der Maaten L, Hinton G. Visualizing data using t-SNE. Journal of machine learning research. 2008;9(11).
